# Integrative single-cell transcriptomic analyses reveal the cellular ontological and functional heterogeneities of primary and metastatic liver tumors

**DOI:** 10.1186/s12967-024-04947-9

**Published:** 2024-02-27

**Authors:** Menghui Gui, Shilin Huang, Shizhou Li, Yuying Chen, Furong Cheng, Yulin Liu, Ji-ao Wang, Yuting Wang, Rui Guo, Yiming Lu, Pengbo Cao, Gangqiao Zhou

**Affiliations:** 1 School of Public Health, Nanjing Medical University, Nanjing, 211166 People’s Republic of China; 2 State Key Laboratory of Medical Proteomics, National Center for Protein Sciences at Beijing, Beijing Institute of Radiation Medicine, 27 Taiping Road, Beijing, 100850 People’s Republic of China; 3 Department of Medical Oncology, Guangxi Medical University Cancer Hospital, Nanning, 530021 People’s Republic of China; 4 Hengyang Medical College, University of South China, Hengyang, 421001 People’s Republic of China; 5 Institute of Life Science and Green Development, College of Life Sciences, Hebei University, Baoding, 071002 People’s Republic of China; 6 Mudanjiang Medical College, Mudanjiang, 157011 People’s Republic of China; 7 College of Chemistry & Environmental Science, Hebei University, Baoding, 071002 People’s Republic of China

**Keywords:** Primary liver tumors, Metastatic liver tumors, Single-cell RNA sequencing, Tumor microenvironment, Ontological heterogeneity, Functional heterogeneity

## Abstract

**Background:**

The global cellular landscape of the tumor microenvironment (TME) combining primary and metastatic liver tumors has not been comprehensively characterized.

**Methods:**

Based on the scRNA-seq and spatial transcriptomic data of non-tumor liver tissues (NTs), primary liver tumors (PTs) and metastatic liver tumors (MTs), we performed the tissue preference, trajectory reconstruction, transcription factor activity inference, cell–cell interaction and cellular deconvolution analyses to construct a comprehensive cellular landscape of liver tumors.

**Results:**

Our analyses depicted the heterogeneous cellular ecosystems in NTs, PTs and MTs. The activated memory B cells and effector T cells were shown to gradually shift to inhibitory B cells, regulatory or exhausted T cells in liver tumors, especially in MTs. Among them, we characterized a unique group of TCF7+ CD8+ memory T cells specifically enriched in MTs that could differentiate into exhausted T cells likely driven by the p38 MAPK signaling. With regard to myeloid cells, the liver-resident macrophages and inflammatory monocyte/macrophages were markedly replaced by tumor-associated macrophages (TAMs), with TREM2+ and UBE2C+ TAMs enriched in PTs, while SPP1+ and WDR45B+ TAMs in MTs. We further showed that the newly identified WDR45B+ TAMs exhibit an M2-like polarization and are associated with adverse prognosis in patients with liver metastases. Additionally, we addressed that endothelial cells display higher immune tolerance and angiogenesis capacity, and provided evidence for the source of the mesenchymal transformation of fibroblasts in tumors. Finally, the malignant hepatocytes and fibroblasts were prioritized as the pivotal cell populations in shaping the microenvironments of PTs and MTs, respectively. Notably, validation analyses by using spatial or bulk transcriptomic data in clinical cohorts concordantly emphasized the clinical significance of these findings.

**Conclusions:**

This study defines the ontological and functional heterogeneities in cellular ecosystems of primary and metastatic liver tumors, providing a foundation for future investigation of the underlying cellular mechanisms.

**Supplementary Information:**

The online version contains supplementary material available at 10.1186/s12967-024-04947-9.

## Background

Liver cancer can be divided into two categories based on its source: primary liver cancer (PLC) and secondary liver cancer. Although the liver is the sixth most common site for primary cancer, liver cancer in situ is the fourth leading cause of cancer-related deaths worldwide [1]. Hepatocellular carcinoma (HCC) accounts for 80–90% of PLCs, and it occurs almost exclusively in the setting of chronic inflammation, which markedly influences hepatic microenvironment [2]. Furthermore, the liver is a common site to metastasis due to its anatomical location and immunosuppressive environment, which make it a favorable site for tumor cells [3]. Patients with metastatic liver tumors mostly originated from tumors of the gastrointestinal tract (e.g., colorectum, esophagus, stomach, and pancreas) have reduced 5-year survival rate and quality of life [4]. In the microenvironment of metastatic tumors, tumor cells not only recruit inflammatory and immune cells but also rely on the involvement of stromal cells, such as fibroblasts [5]. The primary tumor and metastatic tumor microenvironments in the liver may exhibit high heterogeneity, such as cellular composition, spatial distribution of tumor cells, blood supply and angiogenesis ability. These differences can contribute to varying sensitivity and/or resistance to treatments between the primary and metastatic tumors. Therefore, a better understanding of the complex cellular and molecular characteristics of microenvironment that distinguish the primary and metastatic liver tumors will provide valuable insights into the regulatory mechanisms of tumor microenvironment (TME) and will be helpful in developing novel immunotherapy strategies for liver cancers.

Multiple single-cell RNA sequencing (scRNA-seq) studies have investigated separately the cellular microenvironment of primary or metastatic tumors in the liver [6, 7]. However, these studies had not provided a comprehensive comparation of the characteristics of them. Here, based on scRNA-seq data from 8 non-tumor liver tissues (NTs), 10 primary liver tumors (PTs) and 12 metastatic liver tumors (MTs), which consist of 128,118 high-quality cells, we constructed a multicellular ecosystem map of primary and metastatic tumors in the liver. Through comparing the molecular characteristics of multiple cell types, we identified distinct roles of several important myeloid and lymphoid cells in the progression of PTs and MTs. Additionally, inter-cellular interaction analyses emphasized the pivotal roles of malignant hepatocytes and fibroblasts in shaping the TME of primary and metastatic tumors in the liver, respectively. Taken together, this comprehensive landscape of multicellular ecosystem of primary and metastatic liver tumors provides valuable insights for further discriminating the cellular mechanisms and developing potentially effective immunotherapy strategies for this malignancy.

## Materials and methods

### Participants and scRNA-seq data

This study includes a cohort consisting of 10 patients with primary HCC, as well as four cohorts consisting of 8 patients with colorectal cancer (CRC) and 4 patients with pancreatic cancer (PC), all of whom had the metastatic liver tumors. The patients with primary HCC were enrolled between July 2018 and December 2018 at the Chinese PLA General Hospital (Beijing, China). The diagnosis of HCC and the inclusion and exclusion criteria for the patients were described in detail previously [8]. Briefly, all the HCC patients were newly diagnosed, pathologically confirmed, and proved not having other types of cancer. The scRNA-seq data of 10 primary tumor tissues and 8 non-tumor liver tissues were derived from our previous study [9]. Detailed pathological and clinical information of these patients were listed in Additional file 2: Table S1.

The scRNA-seq data of metastatic liver tumors from the CRC patients were derived from the GEO database (GSE178318 [n = 6] and GSE225857 [n = 2] [10]). The GSE178318 dataset was derived from a cohort consisting of six colorectal liver metastases. Among the patients, patients CRC03, CRC05, and CRC06 have received preoperative chemotherapy, and the others were treatment-naïve. The GSE225857 dataset was derived from a cohort consisting of two colorectal liver metastases. The scRNA-seq data of liver metastases from the pancreatic cancer patients were also derived from the GEO database (GSE162708 [n = 1] [11] and GSE154778 [n = 3] [12]). The patient of GSE162708 received a pancreaticoduodenectomy and partial hepatectomy without any anti-cancer treatment prior to operation. Detailed pathological and clinical information of patients were listed in Additional file 2: Table S1. All the scRNA-seq data collected above were generated using the 10× Genomics Chromium platform.

Additionally, the spatial transcriptome dataset contains a colorectal liver metastases who has received preoperative chemotherapy and/or radiotherapy was downloaded from the GEO database (GSE225857) [10] and another dataset contains two colorectal liver metastases (ST-2: untreated; ST-4: neoadjuvant chemotherapy) [13] was download from The National Omics Data Encyclopedia (https://www.biosino.org/node/project/detail/OEP001756). Detailed pathological and clinical information of patients were listed in Additional file 2: Table S1.

### Pre-processing and quality control of scRNA-seq data

Cell Ranger (version 6.0.2; 10× Genomics, USA) was used to work with the raw fastq data and generate gene count matrices with all default parameter settings (based on the human genome reference set GRCh38). The output filtered gene expression matrices of each sample were analyzed by R software (v.4.2.3) with the Seurat (v.4.4.0) package. To filter out low-quality cells and doublets (i.e., one cell expresses two different classical cells’ makers), for each sample, the cells with the number of unique molecular identifiers (UMIs) fewer than 200, or the number of expressed genes over 5,000 or below 200 were removed. To filter out the dead or dying cells, the cells that had over 5% UMIs derived from mitochondrial genome were further removed. In addition, we used the R package DoubletFinder (v.2.0.3) to calculate a doublet score for each cell to remove the potential doublets, with an expected value of 7.6% per 1000 droplets. After quality control, a total of 128,118 high-quality cells from 30 samples were retained for subsequent analyses. Detailed statistic information of patients were listed in Additional file 2: Table S2.

### Integrative analyses of multiple datasets

For dimensionality reduction, the 2000 most variable genes with the highest standardized variance were selected using the ‘FindVariableFeatures’ function and method ‘vst’ in Seurat, and then these genes were processed by principal component analysis (PCA). Next, the ‘RunHarmoy’ function in Harmony package (v.1.1.0) [14] was used for performing sample batch correction. The FindNeighbors’ and ‘FindClusters’ were used for determining cell clusters. In brief, the top 30 principal components (PCs) were selected to construct the shared nearest-neighbor (SNN) graph by calculating the neighborhood overlap between every cell and its nearest neighbors, and a SNN modularity optimization-based clustering algorithm was adopted for cell-clustering. To visualize different cell clusters in a 2-dimension (2D) graph, UMAP dimensional reduction was performed based on the top 30 PCs using the ‘RunUMAP’ function according to the above steps.

### Cell clusters annotation

To identify the marker genes for each cell cluster, we contrasted cells from a cluster to all the other cells of that cluster using the ‘FindMarkers’ function of Seurat, which identifies the differentially expressed genes (DEGs) between two groups of cells using a Wilcoxon rank-sum test. *P* values were then corrected using Bonferroni correction based on the total number of genes in the dataset. Marker genes were required to have an adjusted *P* value < 0.05, an average expression level in that cluster that was at least twofold higher than the average expression level in the other clusters. Then, the annotation of each cell cluster was confirmed by the expression of canonical marker genes. In detail, the T/NK cells were identified by expression of *CD3D*, *CD3E* and *NKG7*; memory B cells by *CD79A* and *MS4A1*; plasma cells by *IGHG1*, *JCHAIN* and *MZB1*; monocytes and macrophages by *CD68*, *CD163*, *CD14* and *LYZ*; dendritic cells by *CD74*, *CLEC9A* and *CD1C*; fibroblasts by *COL1A1*, *ACTA2* and *TAGLN*; endothelial cells by *VWF*, *PLVAP* and *CLDN5*; and epithelial cells by *EPCAM*, *KRT8* and *KRT18*.

### Pathway enrichment analyses

To explore the functional relevance of the candidate cell clusters, pathway enrichment analyses of the DEGs was performed using the R package clusterProfiler (v.4.2.2) [15] based on the gene sets of Gene Ontology (GO) or HALLMARK derived from the MsigDB (v2022.1.Hs; www.broadinstitute.org/gsea/msigdb). The enriched terms with adjusted *P* < 0.05 were considered to be statistically significant. The online Metascape tool (https://metascape.org/) was used for functional enrichment analyses of DEGs in fibroblasts. The detailed results of pathway enrichment were listed in Additional file 2: Table S4.

### Tissue enrichment analyses

To quantify the enrichment of cell types across different tissue groups, we compared the observed and expected cell numbers for each cluster in each tissue group according to the following formula as we previously described [16]: Ro/e = (Observed/Expected), where the expected cell numbers of cell types in a given tissue were calculated from the Chi-square test. We assumed that one cluster was enriched in a specific tissue if Ro/e > 1.

### Definition of cell-type or signature scores

We used the signature scores to evaluate the degree to which an individual cell type expressed a certain pre-defined expression gene set [17]. The cell signature scores were calculated by using the ‘AddModuleScore’ function in Seurat with default settings. The B cell receptor signaling pathway (GO:0050853), 22 B cell immune activation related-genes (*CD82*, *CD83*, *CLECL1*, *CLEC2B*, *CLEC2D*, *ACTB*, *CCR7*, *CORO1A*, *JUNB*, *CD74*, *GPR183*, *CD55*, *EZR*, *HLA-DPB1*, *HLA-DRB1*, *LAPTM5*, *HLA-DQB1*, *MS4A1*, *BANK1*, *CD79A*, *FOXP1*, *ELF1*), 18 inflammatory-associated genes (*CCL2*, *CCL3*, *CCL4*, *CCL5*, *CXCL10*, *CXCL9*, *IL1B*, *IL6*, *IL7*, *IL15*, *IL18*, *CXCR4*, *CXCR5*, *CCR5*, *CCR6*, *CCR9*, *CCR10*, *CXCR3*), plasma cell differentiation (GO:0002317), apoptotic signaling pathway (GO:0097190), 12 cytotoxicity-associated genes (*PRF1*, *IFNG*, *GNLY*, *NKG7*, *GZMB*, *GZMA*, *GZMH*, *KLRK1*, *KLRB1*, *KLRD1*, *CTSW* and *CST7*), 11 regulatory-related genes (*IL2RA*, *FOXP3*, *IL2RB*, *IL10RA*, *IKZF2*, *TNFRSF1B*, *TNFRSF4*, *TNFRSF18*, *BATF*, *CTLA4* and *TIGIT*), 6 well-defined exhaustion marker genes (*LAG3*, *TIGIT*, *PDCD1*, *CTLA4*, *HAVCR2* and *TOX*), dendritic cell cytokine production (GO:0002371), dendritic cell chemotaxis (GO:0002407), dendritic cell antigen processing and presentation (GO:0002468), and 34 M2 polarization-related genes (*CD274*, *CD276*, *CD180*, *CX3CR1*, *CXCL1*, *CXCL3*, *CXCL13*, *CCR5*, *CCL1*, *CCL7*, *CCL8*, *CCL13*, *CCL16*, *CCL17*, *CCL18*, *CCL22*, *CCL23*, *CCL24*, *CCR2*, *IL4*, *IL4R*, *IGF1*, *ARG1*, *EGF*, *IL13*, *IL34*, *VEGFA*, *VEGFB*, *VEGFC*, *VEGFD*, *CTSA*, *TGFB2*, *TGFB3*, *CD47*, *MMP14*, *MMP9*, *NCF2*, *CLEC4A*, *CLEC7A*, *FN1*, *LY86*, *WNT7B*, *TNFRSF8*) were used to define the B cell receptor, immune activate, inflammatory and plamsa cell differentiation function of B cells, cytotoxicity, regulatory function and exhaustion of T cells, cytokine production, chemotaxis and antigen processing and presentation of DCs, M2 polarization levels of WDR45B+ TAMs, respectively. Pseudobulking analyses, using independent samples as data points, revealed significant difference in both immune activation, B cell receptor signaling pathway, inflammatory and plasma cell differentiation levels within B cells among the three tissue groups. To compare the differences in pathway activities of an individual cell cluster among NTs, PTs and MTs, we performed gene set variation analyses based on the bulk RNA-seq data from the TCGA-LIHC cohort and the pog570_bcgsc_2020 (designated as MT2020) cohort [18] through the R package GSVA (v.1.46.0) [19] with default settings.

### Single-cell trajectory analyses

Monocle 2 (v.2.20.0) [20] was applied to construct differentiation trajectory of B cells, T cells, macrophages, fibroblasts and epithelial cells, which introduces the strategy of ordering single cells in pseudotime along a trajectory corresponding to a biological process such as cell differentiation and transformation. Briefly, data in the Seurat object was extracted and loaded into the CellDataSet object, and then the genes expressed in more than 10 cells were used for DEG analyses. Further, the significant DEGs were selected as ordering genes; after dimensionality reduction with the ‘DDRtree’ method, the cells ordering and trajectory construction were performed with default parameters.

### TF activity analyses

To identify the key regulatory transcription factors (TFs) in a candidate cell cluster, SCENIC analysis was performed using the pySCENIC [21] package. The required databases for running SCENIC, including the TF database (cisTarget.hg38.mc9nr.feather) and motif annotation database (hgnc.v9.m0.001), were downloaded from the pySCENIC website (https://github.com/aertslab/pySCENIC). The input matrix of pySCENIC was the normalized expression matrix output from Seurat, and the activity of a TF was measured as the Area Under the recovery Curve (AUC) of the genes that are regulated by this TF. To obtain the differentially activated TFs among different cell clusters, the R package limma (v.3.54.2) [22] was used to fit TF-wise linear models and implements empirical Bayes moderated *t*-statistics to determine the statistical significance (Benjamini-Hochberg-adjusted *P* < 0.01). The detailed results of TF activity analyses were listed in Additional file 2: Table S6.

### Cell–cell interaction analyses

CellChat (v.1.6.1) [23], containing the ligand–receptor interaction databases for human and mouse, was used to construct the intercellular communication networks between different cell clusters. First, we used the ‘CellChatDB.human’ function in CellChat to evaluate the major signaling inputs and outputs among all cell clusters. Then, the receptors and ligands expressed in more than 10 cells in a candidate cell cluster were chosen for subsequent analyses. The interactions between distinct cell clusters via putative ligand–receptor pairs were visualized using the ‘ggplot2’ in R package. The detailed results of cell–cell interaction analyses were listed in Additional file 2: Table S9.

### Bulk RNA-seq analyses

The Cancer Genome Atlas-liver hepatocellular carcinoma (TCGA-LIHC) dataset (including 374 PTs and 50 NTs) and MT2020 dataset (including 198 MTs) [18] were used for estimations of candidate cell cluster infiltration and assessments of clinical significance. The bulk RNA sequencing and matched clinical information from the TCGA-LIHC cohort were accessed through the TCGA data portal (https://gdc-portal.nci.nih.gov/), and the RNA-seq data and matched clinical information of liver metastases from the MT2020 cohort were obtained from the cBioPortal database (https://github.com/cBioPortal/datahub/tree/master/public). After log_2_-transformed, the raw count data were subjected to normalization by ‘scale’ function and removal of batch effect by ‘Combat’ function in sva (v.3.46.0) [24]. Patients with overall survival time less than 30 days were excluded to remove the potential bias related to treatment effects.

### Similarity measurement of cell clusters

To assess the similarity between TCF7+ Tpm cells and each of the pre-defined T cell clusters (Additional file 2: Table S5), and tumor-specific memory T cells (T_TSM_) in tumor-draining lymph nodes (TdLN), we used the ‘AddModuleScore’ function in Seurat to calculate the cell signature scores of pre-defined T cell clusters and TdLN-T_TSM_ cells in all Tpm cells. Then, all the scores were subjected to the ‘cor’ function from the stats (v.3.6.2), and the ‘corrplot’ R package (v.0.92) was used for visualization. For the similarity between fibroblasts and epithelial cells, we extracted the average expression levels of top 50 DEGs of each cluster and calculated their similarities by the ‘cor’ function from the stats. All the correlation coefficients (Rho) and *P* values were determined by Spearman rank correlation analyses.

### Spatial transcriptome data analyses

Seurat was used to work with the Space Ranger output files. The ‘SCTransform’ function in Seurat was used to normalize the data. Then, the scaled matrix was subjected to GSVA analyses for calculating the signature scores of the candidate cell clusters or gene sets in every spot. The reference gene sets used were listed in Additional file 2: Table S10. The ‘SpatialFeaturePlot’ function in Seurat was used to visualize the GSVA signature score.

### Statistical analyses

All data were analyzed and visualized by R (v.4.2.3) in this study. For survival analyses, Kaplan–Meier survival curves were generated using the ‘survival’ in R package, and log-rank test was used to compare the difference in survival curves between two groups. The hazard ratio (HR) and the 95% confidence interval (CI) were computed by using the univariate Cox proportional hazards regression analysis. To evaluate the correlations between fibroblasts and EMT-related genes, as well as the correlations between the ligands, receptors, and cell clusters in TCGA-LIHC and MT2020 cohorts, we computed the signature scores using GSVA based on the EMT-related genes, ligands, receptors, and top 50 DEGs of each cell cluster. Subsequently, the correlations were determined using the ‘cor’ function from the stats package. Wilcox test was used to assess the difference between groups in this study. *P* < 0.05 were considered statistically significant in all statistical tests. Visualization was done using the ‘ggplot2’ R package.

## Results

### scRNA-seq profiling reveals heterogeneous cell composition in primary and metastatic tumors in the liver

**Fig. 1:**
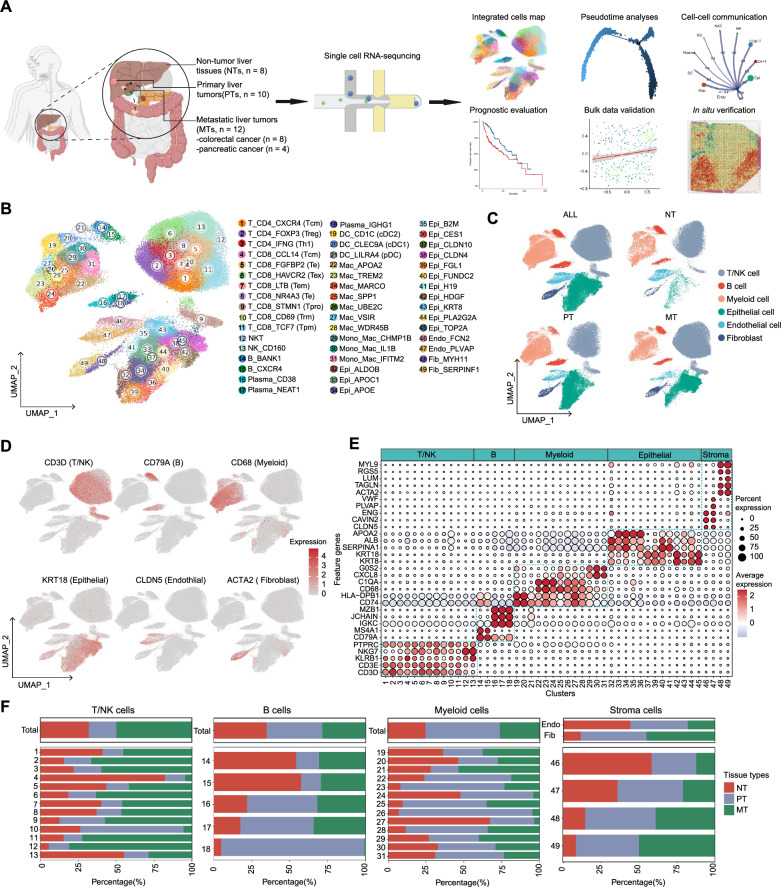
Overview of the single-cell landscape for primary and metastatic liver tumors and non-tumor liver tissues. **A** Schematic diagram of scRNA-seq analysis workflow. The scRNA-seq data generated by 10× Genomics Chromium platform in a total of 8 non-tumor liver tissues (NTs) and 10 primary HCC tumor tissues (PTs) and 12 metastatic liver tumors (MTs) (derived from 8 patients with primary colorectal cancer and 4 patients with pancreatic cancer) were collected for integrated analyses to explore the cellular ontological and functional heterogeneity of primary and metastatic tumors in the liver. The figure was created with biorender.com. **B** Uniform manifold approximation and projection (UMAP) plot showing the transcriptome landscape of 128,118 high-quality cells, which consists 49 cell clusters from 6 major cell types. Cells are colored by clusters. *DC* dendritic cell, *Mac* macrophage, *Mono* monocyte, *Epi* epithelial cell, *Endo* endothelial cell, *Fib* fibroblast. **C** UMAP plot showing the 6 major cell types in NTs, PTs and MTs, colored by cell types. **D** Feature plots of selected typical canonical markers of each major cell type. **E** Dotplot showing the percentage of expressed cells and average expression levels of canonical marker genes of the 49 cell clusters. **F** Clustering of cellular components and their composition proportions in tumor microenvironment (TME) in NTs, PTs and MTs, colored by tissue types

To explore the transcriptomic states of individual cells in both primary and metastatic tumors in the liver, we previously performed scRNA-seq using the 10× Genomics platform in 10 primary liver tumors (PTs) and 8 matched non-tumor liver tissues (NTs) [9] (Additional file 2: Table S1). Besides, we obtained a scRNA-seq dataset profiled by 10× Genomics platform consisting of 12 metastatic liver tumors (MTs) from another four studies, including eight cases of colorectal cancer [7, 10] and four cases of pancreatic cancer [11, 12] (Additional file 2: Table S1). After integrating all these scRNA-seq datasets and quality control (Additional file 1: Fig. S1A, “Materials and methods” and Additional file 2: Table S2), we obtained the transcriptome data of a total of 128,118 single cells. Through performing dimension reduction analyses by uniform manifold approximation and projection (UMAP) (Fig. 1A), a total of 49 cell clusters were identified (Fig. 1B) and classified into 6 major cell types by the canonical marker genes: including 13 T and natural killer (NK) cell clusters, 5 B cell clusters, 13 myeloid cell clusters, 14 epithelial cell clusters, 2 endothelial cell clusters and 2 fibroblast clusters (Fig. 1C–E, Additional file 1: Fig. S1B, C and Additional file 2: Table S3).

Next, to compare the composition heterogeneity between these three tissue groups, we elucidated the fraction of each major cell type (epithelial cells were excluded from this analysis due to their significant composition disparity; Additional file 1: Fig. S1D, E). We observed that compared to NTs, PTs show significant depletion of T/NK cells and endothelial cells, and significant enrichment of myeloid cells and fibroblasts (Fig. 1F), in line with the previous studies on HCC [25]. Besides, we found that T/NK cells, myeloid cells and fibroblasts are increased, while endothelial cells are depleted in MTs (Fig. 1F). Together, our scRNA-seq analyses suggest the heterogeneity of TME among NTs, PTs and MTs.

### Memory B cells exhibit inhibitory state in metastatic liver tumors

**Fig. 2:**
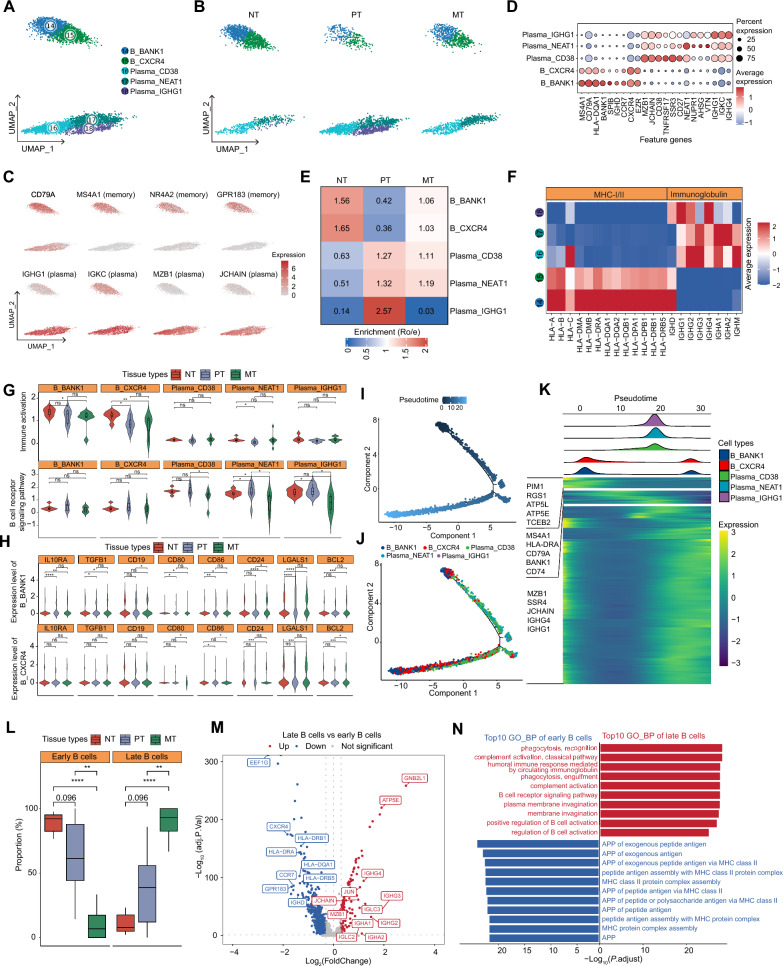
B cell heterogeneity among the primary and metastatic tumors in the liver. **A** UMAP plot of scRNA-seq profile from B cells which are separated into 5 cell clusters. Cells are colored according to different clusters. **B** UMAP plot showing 5 B cell clusters in NTs, PTs and MTs. **C** Feature plots showing the typical markers of memory B cells (top) and plasma cells (bottom). **D** Dotplot showing the percentage of expressed cells and average expression levels of marker genes of the 5 B cell clusters. **E** Tissue prevalence of major B cell clusters estimated by Ro/e scores. Ro/e, the ratios of the observed *versus* expected cell numbers. **F** Heatmap showing the normalized expression (z-score) of B cell function-associated gene sets in each cell cluster. **G** Violin plots showing the immune activation (top) and B cell receptor signaling (bottom) levels of 5 B cell clusters in NTs, PTs and MTs. Wilcox test was used to assess the difference between groups. “**”, “*” and “ns” represent “*P* < 0.01”, “*P* < 0.05” and “not significant”, respectively. **H** Violin plots showing the normalized expression (z-score) of inhibitory-associated genes in memory B cells clusters among three groups. Wilcox test was used to assess the difference between groups. “****”, “***”, “**”, “*” and “ns” represent “*P* < 0.0001”, “*P* < 0.001”, “*P* < 0.01”, “*P* < 0.05” and “not significant”, respectively. **I**–**K** Semi-supervised pseudotime trajectory of B cell clusters inferred by Monocle2. **I** Trajectory is colored by the pseudotime. **J** Trajectory is colored by cell clusters. **K** Ridgeline plot showing the order of appearance of cell clusters in time, colored by cell clusters (top), and the Heatmap showing the fluctuation of genes along the pseudotime (bottom). **L** Box plots showing the proportion changes of early and late B cells in NTs, PTs and MTs, respectively. T-test was used to assess the difference between groups. “****” and “**” represent “*P* < 0.0001” and “*P* < 0.01”, respectively. **M** Volcano plot showing the differential expressed genes (DEGs) between late B cells and early B cells. The red dots represent the statistically significant up-regulated genes, the blue ones represent the down-regulated genes, and the grey ones represent the non-significant genes. **N** Bar plots showing the GO biological processes enriched by the signature genes of late B cells (red) and early B (blue) cells, respectively. *APP* antigen processing and presentation

Next, we first investigated the heterogeneity of B cells among those three tissue groups. B cells were categorized into 5 clusters: 2 memory B cell clusters (B_BANK1 and B_CXCR4) and 3 plasma cell clusters (Plasma_CD38, Plasma_NEAT1 and Plasma_IGHG1) (Fig. 2A–D and Additional file 2: Table S3). Of note, each cell cluster showed distinct tissue preference: the memory B cell clusters were mainly enriched in NTs and MTs, while the plasma cell clusters were mainly found in the PTs and MTs (Fig. 2E and Additional file 1: Fig. S2A).

We then examined the differences in the B cell activation. The memory B cell clusters showed an enhanced immune activation with the upregulation of immuno-stimulatory molecules (e.g., *CD82*, *CD83* and *CLECL*) and MHC molecules while the plasma cell clusters exhibited higher expression of immunoglobulins (Fig. 2F and Additional file 2: Table S3), consistent with the biological processes determined by gene ontology (GO) enrichment analyses (Additional file 1: Fig. S2B and Additional file 2: Table S4). Further, we assessed the functional shift of B cells in PTs and MTs compared to NTs. Surprisingly, despite the fact that memory B cells demonstrated heightened immune activation and pro-inflammatory characteristics compared to plasma cells, their immune activation and inflammatory levels decreased within liver tumors. However, it was observed that the potential of these cells to differentiate into plasma cells significantly increased (Fig. 2G and Additional file 1: Fig. S2C). It is worth noting that there were no or minimal changes of B cell receptor signaling activities for each B cell cluster in PTs, but a markedly reduced activity of B cell receptor signaling was observed in plasma cells in MTs (Fig. 2G). Then, we found that the expression levels of immune inhibitory genes in memory B cells were significantly increased in PTs and MTs, especially in MTs, compared to NTs (Fig. 2H). These findings indicate that while memory B cells generally possess heightened immune activation and inflammation compared to plasma cells, within the context of liver tumors, a paradoxical shift occurs, featuring suppressed inflammation alongside enhanced immunosuppression. Finally, we inferred the developmental trajectories of B cells, and found that memory B cells mainly develop towards plasma cells (Fig. 2I–K), consistent with previous study [26]. Then, we categorized B cells into “early” and “late” stages according to the median pseudotime value. Comparing across tissue types, we observed a decrease in early B cells and an increase in late B cells within liver tumors (PTs and MTs) compared to NTs, particularly in MTs (Fig. 2L). Additionally, we observed that late B cells displayed significant upregulation of plasma cell genes (e.g., *IGHG1*, *JNCAIN*) and downregulation of memory B cell genes (e.g., *GPR183*, *CCR7*) (Fig. 2M). GO enrichment analyses further revealed the activation of the antigen processing and presentation signaling in early cells and B cell receptor signaling in late B cells (Fig. 2N). Collectively, these findings suggest that the B cell developmental trajectory from early to late stages, according to the gene ordering, mirrors the transition from memory B cells to plasma cells.

Furthermore, we assessed the clinical significance of each B cell cluster based on the signature scores derived from the bulk RNA-seq data of PTs from TCGA-LIHC (n = 346) cohort and MTs from MT2020 (n = 198) cohort [18] by using GSVA. None of the B cell cluster with its signature score was significantly associated with the prognosis of patients with HCC; however, it was noteworthy that elevated signature scores of two memory B cell clusters were linked to worse outcomes in patients with metastatic liver tumors (Additional file 1: Fig. S2D–M). Collectively, these data demonstrated that B cells exhibit inhibitory state with pro-tumoral features (e.g., the decreased inflammation and immunosuppression) in liver tumors.

### Convergent CD4+ T and divergent CD8+ T cell compositions and functions in primary and metastatic tumors in the liver

**Fig. 3:**
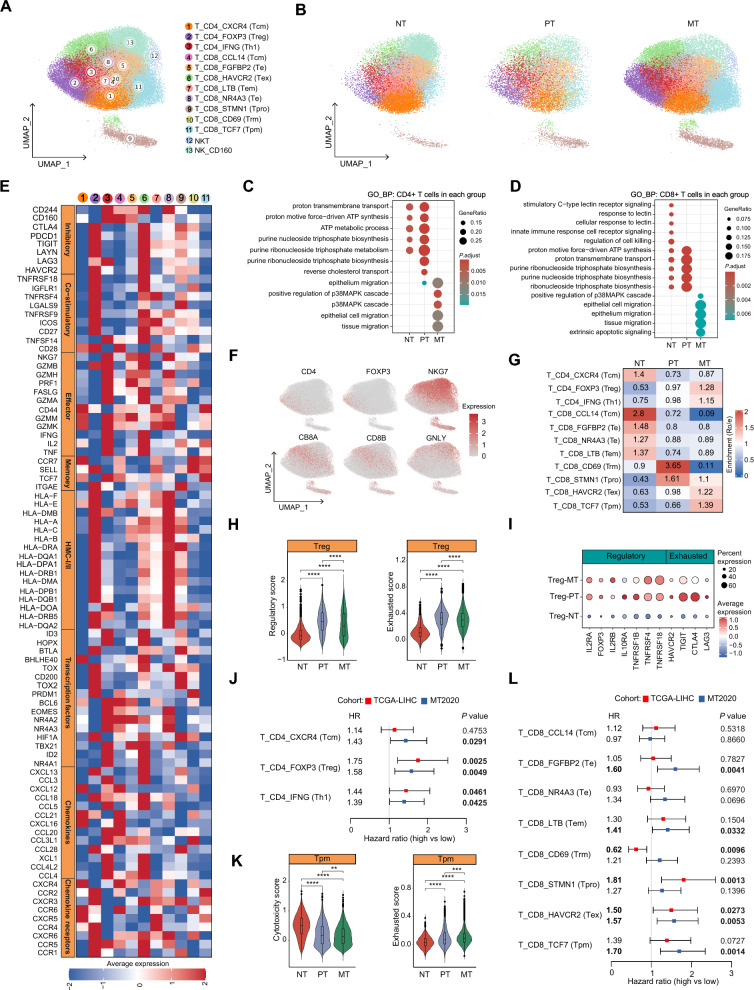
Characterization of the heterogeneous T/NK cell populations in the primary and metastatic liver tumors. **A** UMAP plot of scRNA-seq profile from T/NK cells that are separated into 13 cell clusters. Cells are colored according to different clusters. **B** UMAP plot showing 13 cell clusters in different types of tissues. **C** Dotplot showing GO biological processes enriched by the highly expressed genes of CD4+ T cells in NTs, PTs and MTs, respectively. The dot size indicates the ratios of the enriched genes in the GO term *versus* all highly expressed genes; and the dot color indicates the *P* values determined by Hypergeometric distribution test. **D** Dotplot showing GO biological processes enriched by the highly expressed genes of CD8+ T cells in NTs, PTs and MTs. Dot size indicates the ratios of the enriched genes in the GO term *versus* all highly expressed genes; and dot color indicates the *P* values determined by Hypergeometric distribution test. **E** Heatmap showing the normalized expression (z-score) of T cell function-associated gene sets in each T cell cluster. **F** Feature plots showing the typical markers of CD4+ T cells, CD8+ T cells and NK cells. **G** Tissue prevalence of T cell clusters estimated by Ro/e scores. **H** Violin plots showing the regulatory and exhausted scores of FOXP3+ Treg across different types of tissues. Wilcox test was used to assess the difference between groups. “****” represents “*P* < 0.0001”. **I** Dotplot showing the percentage of expressed cells and average expression levels of regulatory and exhaustion-related genes of Treg in three types of tissues. **J** Forest plot showing the prognostic values of each CD4+ T cell cluster infiltration in the primary HCC cohort (TCGA-LIHC, n = 346) and the metastatic liver tumors cohort (MT2020, n = 198). The CD4+ T cell cluster infiltration was estimated based on the top 50 gene expression signatures of each cell cluster by GSVA. The median GSVA score was used to categorize patients into “high” and “low” groups. The hazard ratios (HRs) with 95% confidence intervals (CIs), and *P* values were determined by univariate Cox proportional hazards regression analyses. **K** Violin plots showing the cytotoxicity and exhaustion scores of TCF7+ progenitor-like memory CD8+ T cells (Tpm) across different types of tissues. Wilcox test was used to assess the difference between groups. “****”, “***” and “**” represent “*P* < 0.0001”, “*P* < 0.001” and “*P* < 0.01”, respectively. **L** Forest plot showing the prognostic values of each CD8+ T cell cluster infiltration in the primary HCC cohort (TCGA-LIHC; n = 346) and the metastatic liver tumors cohort (MT2020; n = 198). The CD8+ T cell cluster infiltration was estimated based on the top 50 gene expression signatures of each cell cluster by GSVA. The median GSVA score was used to categorize patients into “high” and “low” groups

We next characterized the transcriptional properties of the T/NK lymphoid cells. Unsupervised clustering of T/NK lymphoid cells resulted in 13 cell clusters, including 3 CD4+ T cell clusters, 8 CD8+ T cell clusters, one NK cell cluster and one NK-like T cell cluster (Fig. 3A, B and Additional file 1: Fig. S3A). All these clusters were shared among NTs, PTs and MTs. Group-specific GO enrichment analyses showed that there are only slight functional changes for CD4+ T and CD8+ T cells in PTs compared to those in NTs; however, marked changes were observed in MTs compared to either NTs or PTs, especially represented by the cellular processes relevant to the activation of p38 MAPK cascade (Fig. 3C, D and Additional file 2: Table S4). It has been found that deletion of p38 increased multiple phenotypic qualities of effective anti-tumor T cells, e.g., T cell expansion, memory, reactive oxygen species (ROS) and genomic stress (γH2AX) [27]. Pre-conditioning T cells with a p38 inhibitor enhances anti-tumor efficacy of adoptive immunotherapy [27]. Therefore, the activation of p38 MAPK signaling pathway in T cells from MTs may be a pivotal determinant for the tumor immune escape. We then performed a detailed functional definition of all T cells based on the function-related genes in pre-defined T cells [9], including inhibitory receptors, co-stimulatory molecules, effector molecules, memory molecules, antigen presentation-associated molecules, key transcription factors, and chemokines and chemokines receptors. According to the expression levels of canonical marker genes, we classified CD4+ T cells into three categories, including central memory (Tcm: T_CD4_CXCR4), regulatory (Treg: T_CD4_FOXP3) and helper type 1 CD4+ T cells (Th1: T_CD4_IFNG), and classified CD8+ T cells into 7 types, including effector (Te: T_CD8_FGFBP2 and T_CD8_NR4A3), central memory (Tcm, T_CD8_CCL14), effector memory (Tem: T_CD8_LTB), tissue-resident memory (Trm: T_CD8_CD69), progenitor-like memory (Tpm: T_CD8_TCF7), exhausted (Tex: T_CD8_HAVCR2) and proliferative CD8+ T cells (Tpro: T_CD8_STMN1) (Fig. 3E, F). Interestingly, these cell clusters exhibited different tissue preferences: CD4+ Tcm was depleted while Treg and Th1 were enriched in PTs and MTs; CD8+ Tcm, Tem and Te showed enrichment in NTs, CD8+ Trm and Tpro were mainly found in PTs, while CD8+ Tex and Tpm were enriched in MTs (Fig. 3G).

We went on to explore the functional changes of CD4+ T cells in the three groups. The results demonstrated that all three CD4+ T cell clusters, especially the Tregs, in PTs and MTs exhibit higher regulatory and exhausted features than those in NTs (Fig. 3H and Additional file 1: Fig. S3B). Specifically, the expression levels of the regulatory-associated genes (e.g., *IL2RA*, *FOXP3* and *TNFRSF4*) and exhausted-related genes (e.g., *CTLA4*, *TIGIT* and *HAVCR2*) were increased in both PTs and MTs, indicating an immunosuppressive role of Tregs in tumors (Fig. 3I). Further, we explored the clinical significance of CD4+ T cell clusters in PTs from the TCGA-LIHC cohort and MTs from the MT2020 cohort, respectively. Indeed, elevated signature scores of Treg and Th1 were linked to worse outcomes in both patients with primary and metastatic liver tumors (Fig. 3J and Additional file 1: Fig. S3C–H), suggesting the impairment of CD4+ T cells and their promoting effects on immune escape of primary and metastatic tumor cells in the liver.

With regard to CD8+ T cells, continuously decreased cytotoxicity levels and increased exhausted levels were found in all cell clusters in PTs and MTs compared to NTs, suggesting the dysfunction of CD8+ T cells in liver tumors, especially in MTs (Fig. 3K and Additional file 1: Fig. S4A, B). Among them, the four cell clusters (e.g., Tcm, Tem and two Te) enriched in PTs and MTs expressed effector and memory-related genes (Fig. 3E, G, Additional file 1: Fig. S3A and Additional file 2: Table S3). The signature scores of these cell clusters in PTs and MTs had no impact on the patient outcomes, except that CD8+ Te and Tem were associated with poor prognosis in patients with metastatic liver tumors (Fig. 3L and Additional file 1: Fig. S4C–J). Trm cells were enriched in PTs and almost disappeared in MTs (Fig. 3G). The CD8+ Trm cells enriched in the triple-negative breast cancer (TNBC) tumor tissues offer local tissue protection against TNBC tumor re-challenge and are associated with improved treatment outcomes [28]. Consistent with this, we found that Trm cells are a protective factor for the prognosis of patients with primary liver cancer from the TCGA-LIHC cohort (Fig. 3L and Additional file 1: Fig. S4K, L). Compared to that in NTs, the proportions of Tex and Tpm were mildly increased in PTs and sharply elevated in MTs (Fig. 3G). Interestingly, Tpm cells exhibited a resting state with relatively low levels of the functional genes, either inhibitory receptors or effector molecules (Fig. 3E). Moreover, the cytotoxicity levels were significantly reduced in MTs compared to NTs and PTs, while exhausted levels were significantly elevated (Fig. 3K). The result of Cox proportional hazards regression analyses suggested that although it was not related to the survival of patients with primary liver tumors, it was the CD8+ T cell subset most significantly associated with adverse prognosis of patients with metastatic liver tumors (Fig. 3L and Additional file 1: Fig. S4O, P).

Together, our findings suggest that CD4+ T cells may have similar functions in both primary and metastatic liver tumors. However, CD8+ T cells showed significant heterogeneity between the two groups, especially the TCF7+ CD8+ Tpm cells. These cells not only exhibit a significant increase in the proportion in metastatic liver tumors but also pose a dysfunction and exhaustion state, which may facilitate the progression of metastatic liver tumors.

### TCF7+ memory CD8+ T cells exhibit unique pro-tumoral characteristics

**Fig. 4:**
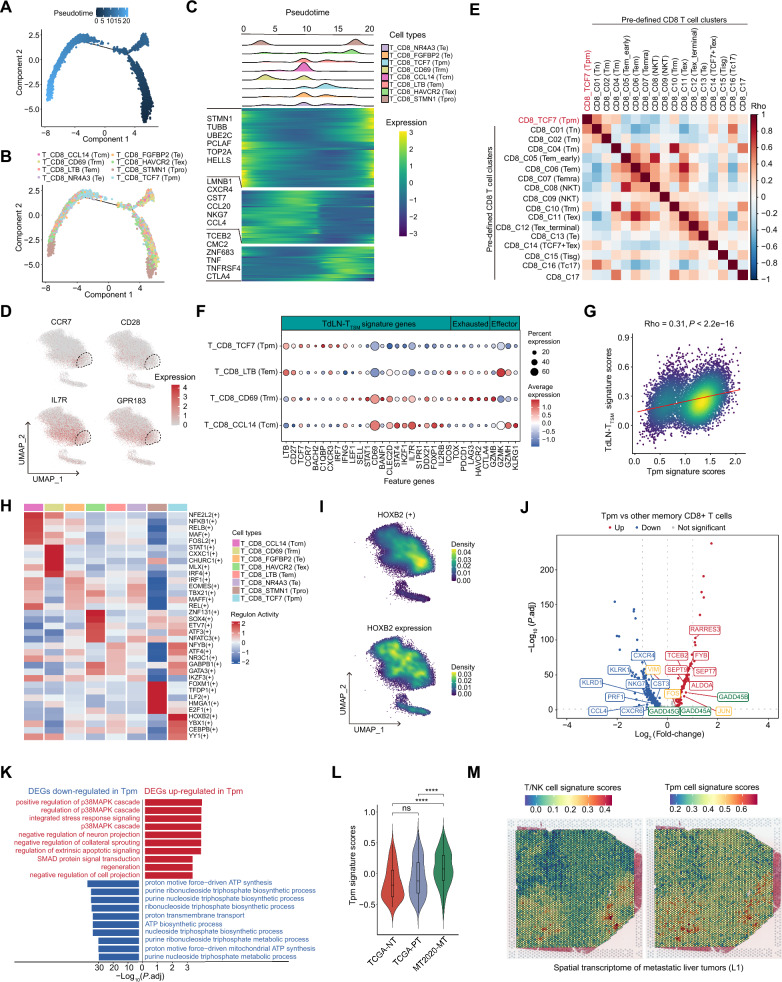
TCF7+ progenitor-like memory CD8+ T cells exhibit immunosuppressive characteristics in metastatic liver tumors. **A**–**C** Semi-supervised pseudotime trajectory of CD8+ T cell clusters inferred by Monocle2. Trajectory is colored by the pseudotime (**A**) or cell clusters (**B**). **C** Ridgeline plot showing the order of appearance of cell clusters in time colored by cell clusters (top), and the heatmap showing fluctuation of genes along the pseudotime (bottom). **D** Feature plots of the typical memory marker genes in each of CD8+ T cell clusters. The TCF7+ Tpm cells are circled by dashed lines. **E** Heatmap of pair-wise correlation coefficients (Spearman correlation analyses) showing the similarity of signature scores between TCF7+ Tpm and pre-defined CD8+ T cell clusters. **F** Dotplot showing the percentage of expressed cells and average expression levels of TdLN-T_TSM_ marker genes, exhaustion, and effector-related genes among 4 CD8+ memory T cell clusters. TdLN-T_TSM_, tumor-draining lymph nodes (TdLN) tumor specific memory T cells (T_TSM_). **G** Scatter plot showing the correlation of signatures scores between TCF7+ Tpm and TdLN-T_TSM_ in TCF7+ CD8+ T cells based on scRNA-Seq data. **H** Heatmap showing the average activities of the top 5 significant transcription factors (TFs) identified by SCENIC in each CD8+ T cell cluster. **I** Feature plots showing the activities of TCF7+ Tpm-specific transcription factor HOXB2 (top) and the expression levels of HOXB2 (bottom). **J** Volcano plot showing the differential expressed genes (DEGs) between TCF7+ Tpm and other memory CD8+ T cells. The red dots represent the statistically significant up-regulated genes, the blue ones represent the down-regulated genes, and the grey ones represent the non-significant genes. Of note, the green boxes mark the p38 MAPK cascade-associated genes and the orange boxes indicate the TGF-beta-SMAD-associated genes. **K** Barplot showing the GO biological processes enriched by the highly and lowly expressed genes in TCF7+ Tpm cells compared with other memory CD8+ T cells. The red and blue bars represent the GO biological processes enriched by the up-regulated genes and down-regulated genes, respectively. **L** Violin plots showing the GSVA scores of TCF7+ CD8+ Tpm cells signature genes across NTs and PTs from the TCGA-LIHC cohort (NTs, n = 50; PTs, n = 374) and MTs from the MT2020 cohort (n = 198). Wilcox test was used to assess the difference between groups. “****” and “ns” represent “*P* < 0.0001” and “not significant”, respectively. **M** Spatial distribution of the whole T/NK cells and TCF7+ Tpm cells in a metastatic liver tumor (L1) determined by the spatial transcriptomic data (GSE225857)

Next, we sought to explore the differentiation trajectories among these heterogeneous CD8+ T cell subpopulations by using Monocle2 [20]. The results showed that the differentiation peak of Tpm cells emerges between the memory T and Tex cells (Fig. 4A–C). It has been demonstrated that exhausted T cells were derived from memory T cell precursors rather than terminal effector T cells in chronic viral infection and cancer, i.e., sustained or disrupted T cell activation signals [29]. Therefore, we speculated that Tpm cells may be the memory T cells which are induced towards exhaustion.

To this end, we initially examined the expression of memory T cell markers (e.g., *CCR7*, *CD28*, *IL7R* and *GPR183*) in the CD8+ T cell clusters. The results showed that Tpm exhibits relatively high levels of these markers (Fig. 4D). Furthermore, we collected the characterized CD8+ T cell clusters that were pre-defined by a previous study [30] (Additional file 2: Table S5), and conducted similarity analyses between the molecular characteristics of the Tpm and these cell clusters. The results aligned with our expectations, indicating a strong association between Tpm and the naïve, memory and Tex cells (Fig. 4E). Therefore, we suspected that Tpm represents a distinct group of progenitor-like memory T cells. Tumor-specific memory T cells (T_TSM_) in tumor-draining lymph nodes (TdLN) can continuously replenish exhausted CD8+ T cells in tumor tissue [31]. Interestingly, Tpm cells, rather than the other memory CD8+ T cells, showed a similar transcriptional signature with TdLN-T_TSM_ (Fig. 4F). Additionally, the cell type signature correlation analyses suggested that Tpm cells exhibit similar properties with TdLN-T_TSM_ (Fig. 4G), suggesting that Tpm is a novel type of tumor-specific memory T cells migrated from the TdLN, which may be interfered and induced to exhaustion, facilitating the immune escape of metastatic cancer cells in the liver.

Further, we tried to explore the function relevance of Tpm cells. First, we investigated the potential driver transcription factors (TFs) underlying the differentiation trajectories by using SCENIC [21]. Distinct sets of TFs were activated among the heterogeneous CD8+ T cell subpopulations, and three TFs (*HOXB2*, *FOS* and *CEBPB*) were highly activated in Tpm cells (Fig. 4H, I and Additional file 2: Table S6). The high expression level of Homeobox B2 (HOXB2) is substantially associated with CD8+ T cell immune infiltration [32]; however, its role in modulation of T cells’ function remains unknown. We then examined the transcriptome differences between Tpm cells and the other CD8+ memory T cells. This analysis revealed significant upregulation of T cells dysfunction-associated genes (*RARRES3* and *ALODA*) [33, 34] and downregulation of effector-associated genes (e.g., *CST3*, *NKG7* and *PRF1*) in Tpm (Fig. 4J and Additional file 2: Table S7). GO enrichment analyses revealed the activation of the immunosuppressive p38 MAPK signaling in Tpm cells (Fig. 4J, K and Additional file 2: Table S4). In addition, bulk transcriptomic data indicated that the signature scores of Tpm cells are significantly increased in tumor tissues, especially in MTs (Fig. 4L). Spatial transcriptome (ST) analyses of three metastatic liver tumors (preoperative chemotherapy: L1, ST-P4 and treatment naïve: ST-P2) also confirmed that there is a large infiltration of Tpm cells in MTs (Fig. 4M and Additional file 1: Fig. S4Q, R). Taken together, these results indicate that the immunosuppressive signature of Tpm cells is likely driven by the p38 MAPK signaling pathway, suggesting the applicability of targeted therapeutic strategies in the treatment of liver metastases.

### Tumor-associated macrophages exert immunosuppressive and metastasis-promoting effects

**Fig. 5:**
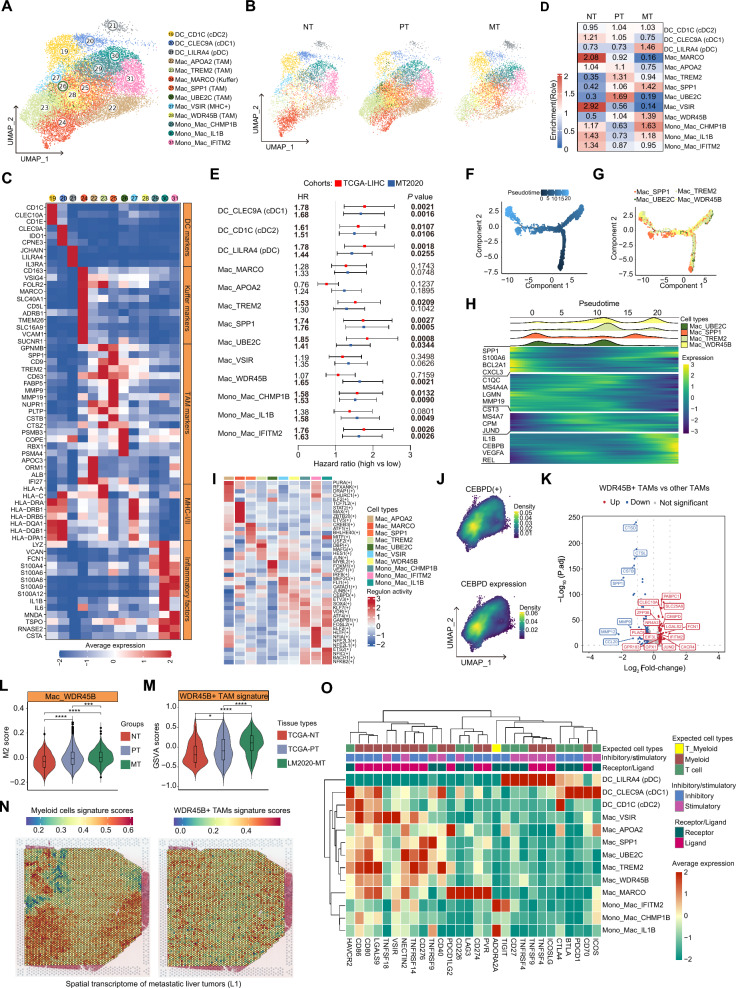
Characterization of the heterogeneity of myeloid cells in primary and metastatic tumors in the liver. **A** UMAP plot of scRNA-seq profile from myeloid cells separated into 13 cell clusters. Cells are colored according to different clusters. **B** UMAP plot showing 13 cell clusters in different types of tissues. **C** Heatmap showing the normalized expression (z-score) of myeloid cell function-associated gene sets in each cell cluster. **D** Tissue prevalence of myeloid cell clusters estimated by Ro/e scores. **E** Forest plot showing the prognostic values of each myeloid cell cluster infiltration in the primary HCC cohort (TCGA-LIHC; n = 346) and the metastatic liver tumors cohort (MT2020; n = 198). The HRs with 95% CIs and *P* values were determined by univariate Cox proportional hazards regression analyses. **F**–**H** Semi-supervised pseudotime trajectory of tumor-associated macrophages clusters inferred by Monocle 2. Trajectory is colored by the pseudotime (**F**) or cell clusters (**G**). **H** Ridgeline plot showing the order of appearance of cell clusters in time colored by clusters (top) and the heatmap showing the fluctuation of genes along the pseudotime (bottom). **I** Heatmap showing the average activities of the top 5 significant TFs identified by SCENIC in each macrophage cluster. **J** Feature plots showing the activities of WDR45B+ TAM-specific TF CEBPD (top) and expression levels of CEBPD (bottom). **K** Volcano plot showing the DEGs between WDR45B+ TAMs and the other TAMs. The red dots represent the significantly up-regulated genes, the blue ones represent the down-regulated genes, and the grey ones represent the non-significant genes. **L** Violin plots showing the M2 scores of WDR45B+ TAMs across different types of tissues. Wilcox test was used to assess the statistic difference between groups. “****” and “***” represent “*P* < 0.0001” and “*P* < 0.001”, respectively. **M** Violin plot showing the GSVA scores of WDR45B+ TAMs signature genes across NTs and PTs from the TCGA-LIHC cohort (NTs, n = 50; PTs, n = 374) and metastatic liver tumors from the M2020 cohort (n = 198). Wilcox test was used to assess the difference between groups. “****” and “*” represent “*P* < 0.0001” and “*P* < 0.05”, respectively. **N** Spatial distribution of the whole myeloid cells and WDR45B+ TAMs in a metastatic liver tumor (L1) determined by the spatial transcriptomic data (GSE225857). **O** Heatmap showing the scaled expression levels of a series of immune checkpoint genes in myeloid cell clusters. Genes are grouped as receptor or ligand, inhibitory or stimulatory status and expected major lineage cell types known to express the gene (lymphocyte and myeloid)

We identified a total of 19,043 myeloid cells which were sub-clustered into 13 cell clusters (Fig. 5A, B and Additional file 1: Fig. S5A). Among them, we designated 3 clusters as dendritic cells (DCs), which displayed various features, including cDC1 (DC_CLEC9A), cDC2 (DC_CD1C) and pDC (DC_LILRA4) (Fig. 5C). Interestingly, pDCs were rarely found in NTs and PTs, but abundant in MTs (Fig. 5D), which exhibited suboptimal capabilities of cytokine production and antigen processing and presentation, but higher levels of chemotaxis than those of cDC1 and cDC2 (Additional file 1: Fig. S5B) as previously described [35]. Notably, high signature scores of pDC were associated with poor prognosis in both patients with primary and metastatic liver tumors (Fig. 5E and Additional file 1: Fig. S5C, D).

Due to the diversity and plasticity of macrophages, their heterogeneity and effect on tumor progression remain largely unknown [36]. Here, we classified the macrophages into four major types: liver-resident Kupffer cells, MHC+ macrophages, monocyte-derived inflammatory macrophages (MoMFs) and tumor-associated macrophages (TAMs) (Fig. 5C). Except for TAMs enriched in PTs and MTs, the other types of macrophages were enriched in NTs, especially the Kupffer-derived macrophages (Mac_MARCO) and MHC+ macrophages (Mac_VSIR), and their signature scores had no impact on tumor patient outcomes (Fig. 5C, E and Additional file 1: Fig. S6A, B). Cells in clusters 29, 30 and 31 were MoMFs, which were characterized by high levels of monocyte markers (e.g., *FCN1*, *LYZ* and *VCAN*) and inflammatory genes (e.g., *IL1B*, *IL6*, *S100A9*, *S100A8* and *CXCL3*) (Fig. 5C and Additional file 1: Fig. S5A and Additional file 2: Table S3). The survival analyses showed that high signature scores of most of these cell clusters are associated with poor prognosis in patients with primary or metastatic liver tumors (Fig. 5E and Additional file 1: Fig. S6C, D).

Besides the three types of macrophages mentioned above, four other TAM clusters (e.g., Mac_TREM2, Mac_UBE2C, Mac_SPP1 and Mac_WDR45B) were enriched in tumor tissues, which displayed high heterogeneity among the three groups (Fig. 5C, D). Concordant with their roles in tumor progression [37], the TREM2+ and UBE2C+ TAMs were abundant in PTs compared to NTs and MTs, which was concordant with their prognostic values for predicting poor survival of patients with primary liver tumors rather than metastatic liver tumors (Fig. 5D, E and Additional file 1: Fig. S6E, F). The SPP1+ and WDR45B+ TAMs were abundant in PTs and MTs, especially in MTs, compared to NTs (Fig. 5C, D). Besides the pro-tumoral role of SPP1+ TAMs in primary tumor progression [38], we here found that it also exhibits a prognostic value for patients with MTs, suggesting its pivotal role in metastatic liver tumors (Fig. 5E and Additional file 1: Fig. S6E, F). The MTs-enriched WDR45B+ TAMs were considered as a new group of macrophages, which have not been clearly characterized before. Of note, the signature score of this cluster was not correlated with the prognosis of patients with PTs, but was a risk factor for patients with MTs (Fig. 5E and Additional file 1: Fig. S6E, F), suggesting its specific role in the metastatic liver tumors.

We further explored the functional relevance of WDR45B+ TAMs. By performing trajectory inference on the four TAM clusters, we found that it emerges at the terminal end of differentiation, marked by inflammatory factors (e.g., *IL1B*, *REL*, *CEBPD* and *JAML*) (Fig. 5F–H). The results of TF enrichment analyses suggested that WDR45B+ TAMs are specifically re-programmed by *CEBPD* (Fig. 5I, J and Additional file 2: Table S6), which can potentiate cytokine production and modulates macrophage function [39]. Next, we explored the unique function of WDR45B+ TAMs compared to the other TAMs, and found upregulation of several known tumor-promoting genes (Fig. 5K and Additional file 2: Table S8), such as *LGALS2* and *GPR183* (Ref [40]). Additionally, we found that the M2 polarization levels of WDR45B+ TAMs are significantly increased in PTs and MTs, especially in MTs, compared to NTs (Fig. 5L). Bulk transcriptomic data from the TCGA-LIHC and MT2020 cohorts showed that the WDR45B+ TAM signature scores slightly increase in PTs, but markedly elevate in MTs, compared to that in NTs (Fig. 5M). In addition, the result of ST analyses of three metastatic liver tumors (preoperative chemotherapy: L1, ST-P4 and treatment naïve: ST-P2) also confirmed that there is a large infiltration of WDR45B+ TAMs in MTs (Fig. 5N and Additional file 1: Fig. S6G, H). Together, these findings indicate that this group of cells may facilitate M2-like polarization of macrophages to induce immunosuppressive effect in the metastatic liver tumors.

Myeloid cells have been reported to be an important source of tumor immune checkpoints [41]. Therefore, we further analyzed the expression of immune checkpoints in the myeloid compartment. In general, the expression of immune checkpoints and their ligands exhibit unique patterns in myeloid cell clusters. Interestingly, we observed marked upregulation of a series of immune checkpoints (e.g., *CD86*, *CD80*, *HAVCR2* and *LGALS9*) in most tumor infiltrating myeloid cells, and a unique pattern in TAMs (e.g., *NECTIN2*, *TNFRSF14*, *CD276* and *TNFRSF9*) (Fig. 5O). Collectively, we found that there is a large number of infiltrated immunosuppressive myeloid cells in PTs and MTs, and the SPP1+ and WDR45B+ TAMs may potentiate progression of primary and metastatic tumors in the liver.

### Abundance and function diversity of stromal cells in primary and metastatic liver tumors

**Fig. 6:**
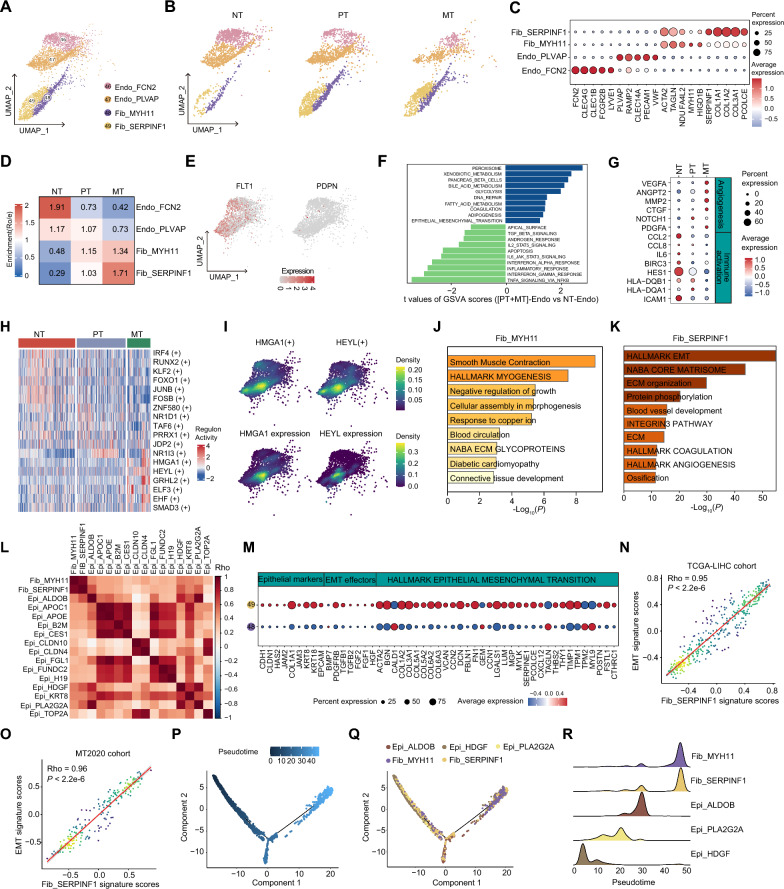
Distinct functions of stromal cells in liver tumors. **A** UMAP plot of scRNA-seq profiles from stroma cells separated into 4 cell clusters. Cells are colored according to different clusters. **B** UMAP plot showing 4 cell clusters in different types of tissues. **C** Dotplot showing the percentage of expressed cells and average expression levels of marker genes of 4 stroma cell clusters. **D** Tissue prevalence of major stroma cell clusters estimated by Ro/e scores. **E** Feature plots showing the expression levels of marker genes for vascular endothelial cells and lymphoid endothelial cells. **F** Differences in hallmark pathway activities scored by GSVA between endothelial cells in tumors (PTs + MTs) and non-tumor liver tissues (NTs). Shown are *t* values from a linear model, corrected for endothelial of origin. **G** Dotplot showing the percentage of expressed cells and average expression levels of angiogenesis- and immune activation-related genes in endothelial cells among three types of tissues. **H** Heatmap showing the activities of enriched transcription factors (TFs) in endothelial cells across three types of tissues. **I** Feature plot showing the activities (top) and the expression levels (bottom) of TFs HMGA1 and HEYL specifically activated in endothelial cells from MTs. **J** Metascape results of the significantly enriched signaling pathways by the genes highly expressed in MYH11+ fibroblasts. **K** Metascape results of the significantly enriched signaling pathways by the genes highly expressed in SERPINF1+ fibroblasts. **L** Heatmap of correlation analyses showing the similarity of signature genes between fibroblast cell clusters and epithelial cell clusters in fibroblasts and epithelial cells of scRNA-seq data. **M** Dotplot showing the percentage of expressed cells and average expression levels of epithelial-to-mesenchymal transition (EMT) effector genes in the two fibroblast clusters. **N**, **O** Scatter plot showing the correlation of signature scores between SERPINF1+ fibroblasts and EMT in PTs from the TCGA-LIHC cohort and MTs from the MT2020 cohort. **P**–**R** Semi-supervised pseudotime trajectory of fibroblasts and high-related epithelial clusters inferred by Monocle 2. **P** and **Q** Trajectory is colored by the pseudotime (**P**) or clusters (**Q**). **R** Ridgeline plot showing the order of appearance of cell clusters in time colored by clusters

We identified two clusters of endothelial cells and two clusters of fibroblasts (Fig. 6A–C). Due to the significant decreases in endothelial cells in both PTs and MTs compared to NTs (Fig. 6D), we explored their functional changes in tumors. First, we performed lineage identification of endothelial cells, and found that almost all endothelial cells express *FLT1* (vascular endothelial cell marker), but not *PDPN* (lymphoid endothelial cell marker) (Fig. 6E). Hallmark pathway enrichment analyses highlighted that metabolic pathways were the most abundant features of tumor-driving endothelial cells, including peroxisomes, xenobiotic metabolism, fatty acid metabolism and glycolysis (Fig. 6F), which are instrumental for angiogenesis [42]. Surprisingly, the most significantly downregulated pathway was involved in inflammatory responses (Fig. 6F). A more detailed analysis revealed downregulation of genes involved in antigen presentation (MHC-I/II), chemotaxis (*CCL2*, *CCL8* and *IL6*) and immune cell homing (e.g., *ICAM1*) in PTs and MTs, compared to NTs (Fig. 6G). Finally, we applied SCENIC to assess which transcription factors underlie the expression differences in endothelial cells between tumors and NTs. The results showed that endothelial cells in tumors are specifically dominated by *HMGA1* and *HEYL* (Fig. 6H, I and Additional file 2: Table S6), both of which have been found as promoters of neoangiogenesis in breast cancers [43, 44]. Together, these results indicate that tumor endothelial cells are remodeled to downregulate their antigen presentation and immune cell homing activities while upregulate their neoangiogenesis capabilities, thus contributing to tumor immunotolerance.

In contrast to endothelial cells, we found that two clusters of fibroblasts significantly increase in PTs and MTs, especially the SERPINF1+ fibroblasts in MTs (Fig. 6D). Pathway enrichment analyses revealed distinct functions of these two clusters: MYH11+ fibroblasts expressed activated fibroblast markers (e.g., *ACTA2*) and smooth muscle cell markers (e.g., *MYH11* and *TAGLN*), which were related to myogenesis and smooth muscle contraction; while SERPINF1+ fibroblasts expressed unique collagens and other extracellular matrix molecules involved in epithelial–mesenchymal transition (EMT) and extracellular matrix (ECM) organization (Fig. 6J, K and Additional file 2: Table S4). Therefore, we hypothesized that SERPINF1+ fibroblasts may derive from the mesenchymal transition of epithelial cells. Signature correlation analyses of fibroblasts and epithelial cells showed that SERPINF1+ fibroblasts display a strong similarity to ALDOB + , HDGF+ and PLA2G2A+ epithelial cells (Fig. 6L). Consistently, we observed that SERPINF1+ fibroblasts show higher expression levels of EMT markers, compared to the MYH11+ fibroblasts (Fig. 6M). Additionally, bulk transcriptomic data from the TCGA-LIHC cohort and MT2020 cohort revealed a consistent strong correlation of characteristic signature scores between SERPINF1+ fibroblasts and EMT (Fig. 6N, O). We further performed trajectory inference of these epithelial cells and fibroblasts. As expected, fibroblasts were found to be terminally differentiated, and the differentiation state of SERPINF1+ fibroblasts were observed to be slightly earlier than that of MYH11+ fibroblasts (Fig. 6P–R), indicating that SERPINF+ fibroblasts might originate from EMT. Taken together, these data indicate remarkable divergences in the abundance and function of stromal cells in primary and metastatic tumors in the liver.

### Malignant hepatocytes and tumor-associated fibroblasts shape the TMEs in PTs and MTs, respectively

**Fig. 7:**
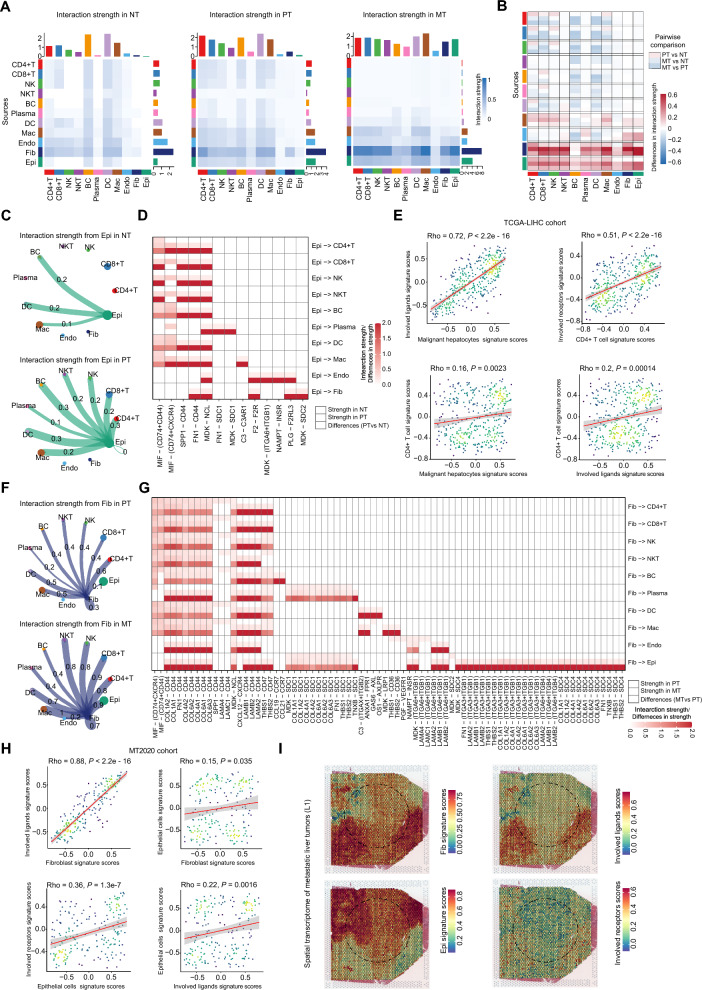
Cell–cell interaction networks in primary liver tumors and metastatic liver tumors. **A** Heatmap illustrating the cell–cell interaction patterns in NTs, PTs, and MTs. **B** Heatmap showing the pair-wise comparison changes of interaction intensities between NTs, PTs, or MTs. **C** Circle plot showing the interaction intensities of the outgoing interactions of malignant hepatocytes with other cell types in NTs (top) and PTs (bottom), respectively. **D** Heatmap showing the enriched outgoing ligand–receptor interaction intensities of hepatocytes with other cell types in NTs and PTs, and their fold changes in PTs compared to NTs. **E** Scatter plots showing the correlations of signature scores between the malignant hepatocytes and the involved ligands, the CD4+ T cells and the involved receptors, the malignant hepatocytes and CD4+ T cells, and the involved ligands and CD4+ T cells in PTs from the TCGA-LIHC cohort (n = 374). **F** Circle plot showing the interaction intensities of the outgoing interactions of fibroblasts with other cell types in PTs (top) and MTs (bottom), respectively. **G** Heatmap showing the enriched outgoing ligand–receptor interaction intensities of fibroblasts with other cell types in PTs and MTs, and their fold changes in MTs compared to PTs. **H** Scatter plots showing the correlations of signature scores between the fibroblasts and the involved ligands, the malignant epithelial cells and the involved receptors, the fibroblasts and malignant epithelial cells, and the involved ligands and malignant epithelial cells in MTs from the MT2020 cohort (n = 198). **I** Spatial distributions of the fibroblasts, the involved ligands, the malignant epithelial cells and the involved receptors in a metastatic liver tumor (L1) determined by the spatial transcriptomic data (GSE225857)

Next, we sought to investigate which type of cells in PTs and MTs shape the tumor microenvironments. We first conducted cell–cell interaction analyses by using CellChat to explore the ligand–receptor (L–R)-mediated intercellular communications for each group. We observed that the weight of cell–cell interactions in PTs and MTs is globally much higher than that in NTs (Fig. 7A and Additional file 2: Table S9). In PTs, fibroblasts dominated the interactions with immune cells. Strikingly, In MTs, fibroblasts dominated the interaction with immune cells and epithelial cells (Fig. 7A). Then, we performed pair-wise comparisons between the three groups based on the intensities of cell–cell interactions. In particular, we found the weight of malignant hepatocytes related L–R interactions dramatically increases in PTs compared to NTs; and fibroblasts related L–R interactions dramatically increases in MTs compared to NTs and PTs (Fig. 7B), suggesting that epithelial cells in PTs and fibroblasts in MTs might play major roles in shaping the microenvironments, respectively.

We next explored the intensity of the outgoing interactions of epithelial cells (e.g., hepatocytes) with other cell types in NTs and PTs, respectively. Of note, compared with NTs, the intensity of immune cells (especially the CD4+ T cells) connected with hepatocytes increased most significantly in PTs (Fig. 7B, C). Malignant hepatocytes in PTs could regulate immune cells, but not stromal cells, through the MIF-(CD74+CD44) and MIF-(CD74+CXCR4) L–Rs (Fig. 7D). When MIF binds to its receptor CD74, it recruits the hyaluronate receptor CD44/CXCR4, forming a complex (CD74/CD44 or CD74/CXCR4) that has been implicated in tumorigenic MIF signaling processes [45]. In addition to the L–Rs involved in the MIF pathway, there were many other L–Rs in the PT microenvironment to promote tumor progression, such as SPP1-CD44 of SPP1 pathway (Fig. 7D). It has been found that SPP1-CD44 and SPP1-PTGER4 interactions mediate the crosstalk between HCC cells and macrophages, which can trigger the polarization of M2 macrophages [46]. We then verified these findings in the PTs from the TCGA-LIHC cohort. The signature scores of the involved ligands were positively correlated with that of the malignant hepatocytes, and a similar positive correlation was observed between the involved receptors and the CD4+ T cells (Fig. 7E). Importantly, positive correlations were also found between the signature scores of CD4+ T cells and malignant hepatocytes or the involved ligands (Fig. 7E). Together, in primary liver tumors, malignant hepatocytes are likely the major drivers, which shape the TME by communicating with immune cells and restraining their anti-tumoral effects.

We then compared the intensity of the outgoing interactions of fibroblasts with other cell types in PTs and MTs (Fig. 7F). Interestingly, we found that compared with PTs, the interaction intensity of fibroblasts connected with malignant epithelial cells increases most significantly in MTs. Indeed, many L–Rs, including collagen/fibronectin/laminin-syndecan/integrin and others, exhibited strong potential interactions between fibroblasts and epithelial cells (Fig. 7G). The activation of these L–Rs was closely related to tumor metastasis [47–49]. Together, fibroblasts can enhance the malignancy of metastatic tumor cells in the liver through a variety of secreted matrix components or THBS1. In addition to malignant epithelial cells, the interaction intensity of fibroblasts with immune cells in MTs markedly increased compared to NTs, but slightly increased compared to PTs (Fig. 7A, G). The L–Rs including the MIF-(CD74+CXCR4) and collagen/fibronectin/laminin-CD44 interactions in MTs may create a tumor immunosuppressive microenvironment. The CD36+ CAFs recruit CD33+ myeloid-derived suppressor cells (MDSCs) via MIF-(CD74+CXCR4) axis [50]. Of note, we also verified these findings in MTs from the MT2020 cohort. The signature scores of the involved ligands were positively correlated with that of the fibroblasts, and a similar positive correlation was observed between the involved receptors and the malignant epithelial cells (Fig. 7H). Importantly, positive correlations were also found between the signature scores of malignant epithelial cells and fibroblasts or the involved ligands (Fig. 7H). In addition, the ST analyses of three metastatic liver tumors (preoperative chemotherapy: L1, ST-P4 and treatment naïve: ST-P2) confirmed the concordant spatial distribution of fibroblasts and the involved ligands, which are close to the edges of malignant epithelial cells, the main source of receptors (Fig. 7I and Additional file 1: Fig. S7A, B). Taken together, fibroblasts could shape the microenvironment of liver metastases by enhancing the malignant epithelial cells of tumor cells and recruiting immunosuppressive cells infiltration.

## Discussion

Numerous studies have recently examined the cellular transcriptomic characteristics of primary liver tumors or metastatic liver tumors [9, 10, 41], respectively. However, there has been no comparative analysis that combines the cellular and molecular characteristics of primary and metastatic tumors in the liver. Hepatocellular carcinoma (HCC), the most prevalent form of primary liver cancer (PLC), representing 80–90% of cases, makes it a suitable choice for studying primary liver tumors, allowing for valuable comparisons with metastatic liver tumors. We here integrated the large-scale scRNA-seq data of non-tumor liver tissues, primary HCC tumors and liver metastatic tumors, providing a high-resolution landscape of cellular heterogeneity in immune and stroma cells, and highlighting the inter-cellular crosstalks.

Our study revealed that the memory B cells primarily exist in non-tumor liver tissues and metastatic tumors, while plasma cells are mainly found in both primary and metastatic liver tumors. The upregulation of IgG and IgA in plasma cells suggests their involvement in class switch recombination. However, this transformation did not affect tumorigenesis and solely represents a maturation process for B cells. Notably, our findings indicated a potential association between inhibitory memory B cells in tumors and the occurrence of metastatic liver tumors. Indeed, tumor-adapted B cells were capable of secreting pathological antibodies targeting the tumor antigen *HSPA4*, thereby promoting breast cancer lymph node metastasis [51]. Altogether, these findings highlight the potential of inhibitory memory B cells as a target for treatment of liver tumors.

Consistent with previous studies, our findings demonstrated a significant increase in regulatory and exhausted T cells in PTs and MTs, while a depletion in effector T cells. The infiltration of exhausted T cells or regulatory T cells in HCCs is associated with adverse effects and significantly impacts prognosis [52, 53]. Notably, we here discovered a previously unexplored cluster of progenitor-like memory CD8+ T cells (e.g., TCF7+ Tpm cells), which exhibits a differentiation state that lies between memory T cells and exhausted T cells. Previous reports have highlighted the presence of unique memory T cells in TME, which may transform into exhausted T cells upon antigen stimulation [31]. Surprisingly, this new cluster of cells showed minimal expression of effector molecules and was specifically correlated with poor prognosis in patients with metastatic liver tumors, rather than those with primary liver tumors. In ovarian cancer ascites, the memory T cells serve as an important supplementary pool of terminal T cells in primary tumors and metastases [54], suggesting the involvement of TCF7+ Tpm cells in metastatic liver tumors. In this study, we found that the transcription factor *CEBPB* and the p38 MAPK cascade pathway are activated in TCF7+ Tpm cells. CEBPB, which acts as a transcriptional repressor of T cell related genes, is phosphorylated by p38 MAPK [55]. CD36 on CD8+ tumor infiltrating lymphocytes (TILs) contributes to T cell dysfunction by facilitating the uptake of oxidized low-density lipoproteins (OxLDL) into T cells. This uptake leads to lipid peroxidation and subsequent activation of p38 MAPK kinase. Interestingly, inhibiting p38 MAPK kinase partially restored the secretion of TNF and IFNγ in the presence of OxLDL [56]. Another study also confirms that p38 inhibition can enhances the secretion of IFNγ and Granzyme-B by T cells in response to TCR stimulation, thereby improving T cell functionality [57]. Therefore, these findings suggest that in metastatic liver tumors microenvironment, memory CD8+ T cells are interfered (e.g., excessive lipid accumulation) and induced exhaustion driven by the p38 MAPK-CEBPB axis. Certainly, it is necessary to further investigate the mechanism by which TCF7+ Tpm exerts immunosuppressive effects in metastatic liver tumors.

In this study, the predominance of myeloid cells in tumor tissues prompted us to elaborate the function of myeloid cells in the tumor microenvironment. Interestingly, pDCs infiltrate massively in MTs. Previous studies have demonstrated that pDCs significantly infiltrate liver cancer tissues, thereby facilitating vascular invasion and lymph node metastases [58]. Therefore, these findings suggest that pDCs provide a prerequisite for tumor metastasis to the liver by creating an immunosuppressive microenvironment. Additionally, among the TAM clusters identified in this study, TREM2+ and SPP1+ TAMs have been previously reported in various tumors [59, 60]. In addition, we here identified a distinct cluster of macrophages (e.g., WDR45B + TAMs) that express high levels of *WDR45B*, which has been shown to play a critical role in autophagosome maturation [61]. Autophagy has been shown to influence the metabolic state within cells and subsequently regulates macrophage M2 polarization [62]. Additionally, compared to the other TAMs, WDR45B+ TAMs exhibited relatively high level of *LGLAS2*, which facilitates M2-like polarization of macrophages through *CSF1* [40]. Based on these findings, we speculate that the increased autophagy levels in WDR45B+ TAMs may direct it towards M2 polarization, thereby facilitating the adaption and progression of metastatic tumors in the liver, which also needs to be confirmed in future study.

We here revealed that the malignant hepatocytes and fibroblasts are the major divers in shaping the cellular microenvironments of primary and metastatic liver tumors, respectively. The intensity of interactions between malignant hepatocytes and immune cells significantly increased in PTs compared with NTs via L–Rs, e.g., MIF-(CD74+CD44), SPP1-CD44, which have been known to be immunosuppressive and pro-angiogenic as previously reported [45, 46]. Numerous studies have shown that CAFs do not exist as separate cells around tumors, but interact with tumor cells to promote tumor growth and survival and maintain their malignant tendencies [63, 64]. Consistently, we here found that fibroblasts have the strongest interactions with malignant epithelial cells via the abundant collagen/fibronectin/laminin-syndecan/integrin interactions in MTs. In addition, we would like to explore in depth how the liver microenvironment recruits various tumor cells to metastasize to it. In this regard, chronic alterations of the hepatic immune microenvironment accompanied by progressive changes of the metabolic profile have been shown to promote and trigger the development of primary liver tumors [65]. Additionally, the apparently tumor-hostile environment of the healthy liver can also be modified acutely and transiently for homing and hosting of metastatic cells originating from extrahepatic malignancies [66]. It is well-known that the liver is a common site for metastasis, particularly after lymph nodes [4], with nearly 50% of CRC patients developing liver metastases, due to the bidirectional gut-liver relationship and the immunosuppressive nature of the liver. The formation of a pre-metastatic niche in the target organ seems to be an essential prerequisite for invasion and dissemination of cancer cells (the ‘seed and soil’ theory [67]). Recent studies have shown that lung fibroblasts facilitate pre-metastatic niche formation by remodeling the local immune microenvironment, thereby promoting breast cancer metastasis [68]. Therefore, these findings indicate that intrahepatic fibroblasts may play a pivotal role in remodeling the liver microenvironment, thereby facilitating the tumor metastasis. Even after the establishment of metastases, fibroblasts persistently contribute to tumor progression and invasion, thereby impacting the features of the microenvironment for the adaptation of metastatic cells. However, the underlying mechanism requires further investigation and exploration.

This study has several limitations that should be acknowledged. Firstly, this study is based on our and multiple published datasets, which may have different standards for diagnosing and treating, leading to variations in the causes of the included patients. Future studies should be conducted enrolled patients according consistent diagnostic and treatment standards. Secondly, the types of metastatic tumors included in this study are limited, and the findings here need to be validated in a wide range of metastatic liver tumors.

## Conclusion

Taken together, the single cell transcriptome atlas provides a comprehensive TME characterization of primary and metastatic tumors in the liver. The systematic study of transcription changes may provide valuable insights for further investigating the biological functions and molecular mechanisms, which will be helpful in developing or improving therapeutic strategies for liver cancers.

### Supplementary Information

**Additional file 1: Figure S1.** Overview of the single-cell atlas from primary and metastatic liver tumors and non-tumor tissues. **Figure S2.** Characteristic heterogeneity of B cells in primary and metastatic liver tumors. **Figure S3.** Ontological and functional changes of CD4+ T cells in primary and metastatic liver tumors. **Figure S4.** Functional changes and clinical relevance of CD8+ T cell signatures in primary and metastatic liver tumors. **Figure S5.** Characteristics of myeloid cells in primary and metastatic liver tumors. **Figure S6.** Clinical relevance and spatial distribution of macrophage signatures in primary and metastatic liver tumors. **Figure S7.** Spatial distributions of the fibroblasts, the involved ligands, the malignant epithelial cells and the involved receptors in metastatic liver tumors.

**Additional file 2: Table S1.** Clinical characteristics of enrolled patients. **Table S2.** Statistics of single-cell RNA sequencing data. **Table S3.** Cell count across patients and tissue types and marker genes of 49 cell clusters. **Table S4.** Pathway enrichment analyses in cell clusters. **Table S5.** The characterized CD8+ T cells pre-defined by a previous study. **Table S6.** Transcription factors activity analyses in this article. **Table S7.** Differentially expressed genes between the TCF7+ CD8+ memory T cells and the other memory CD8+ T cells. **Table S8.** Differentially expressed genes between the WDR45B+ TAMs cells and the other TAMs. **Table S9.** Significantly enriched L–R interactions in NT, PT and MT. **Table S10.** Marker genes of candidate cell types or signatures.

## Data Availability

The processed gene expression data of scRNA-seq of primary HCC tumors and non-tumor liver tissues are available at the GEO database under the accession code GSE149614. The names of the repository/repositories and accession numbers of the publicly available datasets can be found in the article/Additional files.

## Abbreviations

TME: Tumor microenvironment
scRNA-seq: Single-cell RNA sequencing
ST: Spatial transcriptome
NT: Non-tumor liver tissue
PT: Primary liver tumor
MT: Metastatic liver tumor
p38 MAPK: P38 mitogen-activated protein kinase
TAM: Tumor-associated macrophage
PLC: Primary liver cancer
HCC: Hepatocellular carcinoma
CRC: Colorectal cancer
PC: Pancreatic cancer
GEO: Gene Expression Omnibus
TCGA: The Cancer Genome Atlas
LIHC: Liver hepatocellular carcinoma
SNN: Shared nearest-neighbor
DEG: Differentially expressed gene
GO: Gene ontology
TF: Transcription factor
GSVA: Gene set variation analysis
HR: Hazard ratio
CI: Confidence interval
ROS: Reactive oxygen species
Tcm: Central memory T cell
Tem: Effector memory T cell
Trm: Tissue-resident memory T cell
Tpm: Progenitor-like memory T cell
Te: Effector T cell
Tex: Exhausted T cell
Treg: Regulatory T cell
Th1: Helper type 1 T cell
Tpro: Proliferative T cel
TNBC: Triple-negative breast cancer
T_TSM_: Tumor-specific memory T cells
TdLN: Tumor-draining lymph node
DC: Dendritic cell
MOMFs: Monocyte-derived inflammatory macrophage
EMT: Epithelial–mesenchymal transition
ECM: Extracellular matrix
L–R: Ligand–receptor
Mac: Macrophage
Mono: Monocyte
Epi: Epithelial cell
Endo: Endothelial cell
Fib: Fibroblast
