# Supplementary material for: Integrative single-cell transcriptomic analyses reveal the cellular ontological and functional heterogeneities of primary and metastatic liver tumors
Source: J Transl Med. 2024 Feb 27;22:206. doi: 10.1186/s12967-024-04947-9 (PMC10898050; doi:10.1186/s12967-024-04947-9)
Supplement: Supplementary file 1 — Additional file 1: Figure S1. Overview of the single-cell atlas from primary and metastatic liver tumors and non-tumor tissues. Figure S2. Characteristic heterogeneity of B cells in primary and metastatic liver tumors. Figure S3. Ontological and functional changes of CD4+ T cells in primary and metastatic liver tumors. Figure S4. Functional changes and clinical relevance of CD8+ T cell signatures in primary and metastatic liver tumors. Figure S5. Characteristics of myeloid cells in primary and metastatic liver tumors. Figure S6. Clinical relevance and spatial distribution of macrophage signatures in primary and metastatic liver tumors. Figure S7. Spatial distributions of the fibroblasts, the involved ligands, the malignant epithelial cells and the involved receptors in metastatic liver tumors. [file 12967_2024_4947_MOESM1_ESM.docx]

**Additional file 1 Figures:**


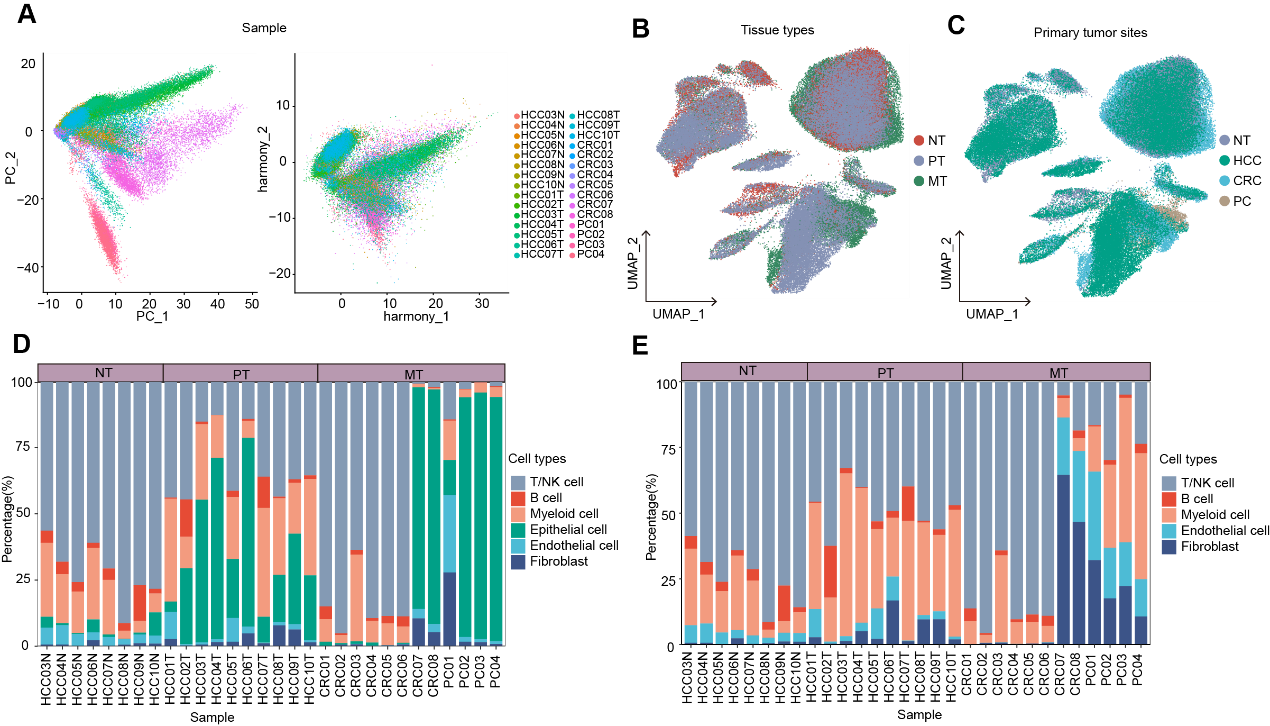


**Fig. S1. Overview of the single-cell atlas from primary and metastatic liver tumors and non-tumor tissues. (A)** Samples distribution in a two-dimensional space by principal component analysis (PCA) before (left) and after (right) the removal of batch effects by using Harmony. **(B)** UMAP plot showing the scRNA-seq profiles in NTs, PTs and MTs. Cells were colored according to the tissue types. **(C)** UMAP plot showing the scRNA-seq profiles in different primary sites of tumors. Cells were colored according to the primary sites of tumors. **(D)** Stacked barplot showing the percentages of major cell types of each sample in NTs, PTs and MTs. Cells were colored according to the cell types. **(E)** Stacked barplot showing the percentages of major cell types (except epithelial cells) for each sample in NTs, PTs and MTs. UMAP, uniform manifold approximation and projection; NT, non-tumor liver tissue; PT, primary liver tumor; MT, metastatic liver tumor.


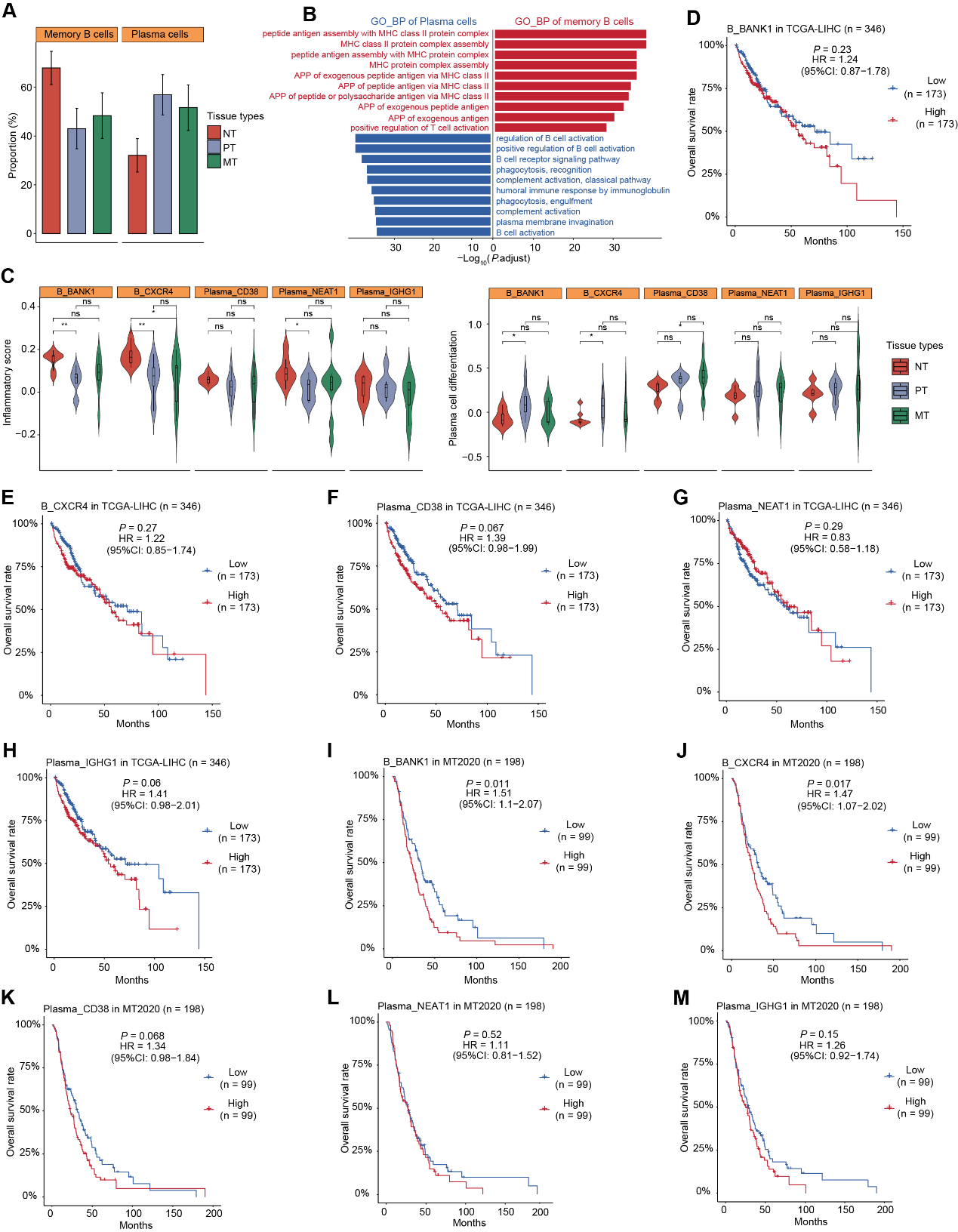


**Fig. S2. Characteristic heterogeneity of B cells in primary and metastatic liver tumors. (A)** Barplot showing the proportion changes of memory B cells and plasma cells in NTs, PTs and MTs, respectively. **(B)** Barplot showing the GO biological processes enriched by the signature genes of memory B cells and plasma cells, respectively. **(C)** Violin plots showing the inflammatory (left) and plasma cell differentiation (right) levels of the 5 B cell clusters in NTs, PTs and MTs. Wilcox test was used to assess the difference between groups. “**”, “*” and “ns” represent “*P* < 0.01”, “*P* < 0.05” and “not significant”, respectively. **(D-H)** Kaplan-Meier survival plots of patients with primary hepatocellular carcinoma from the TCGA-LIHC cohort (n = 346) grouped by the signature scores of each B cell cluster. The cluster infiltration levels were estimated based on the top 50 signature genes of each cell cluster by GSVA. The median GSVA score was used to categorize the patients into “high” and “low” groups. The hazard ratios (HRs) with 95% confidence intervals (CIs), and *P* values were determined by univariate Cox proportional hazards regression analyses. **(I-M)** Kaplan-Meier survival plots of patients with metastatic liver tumors from the MT2020 cohort (n = 198) grouped by the signature scores of each B cell cluster. The cluster infiltration levels were estimated based on the top 50 signature genes of each cell cluster by GSVA. The median GSVA score was used to categorize the patients into “high” and “low” groups. GSVA, gene set variation analysis; NT, non-tumor liver tissue; PT, primary liver tumor; MT, metastatic liver tumor; TCGA-LIHC, The Cancer Genome Atlas-liver hepatocellular carcinoma.


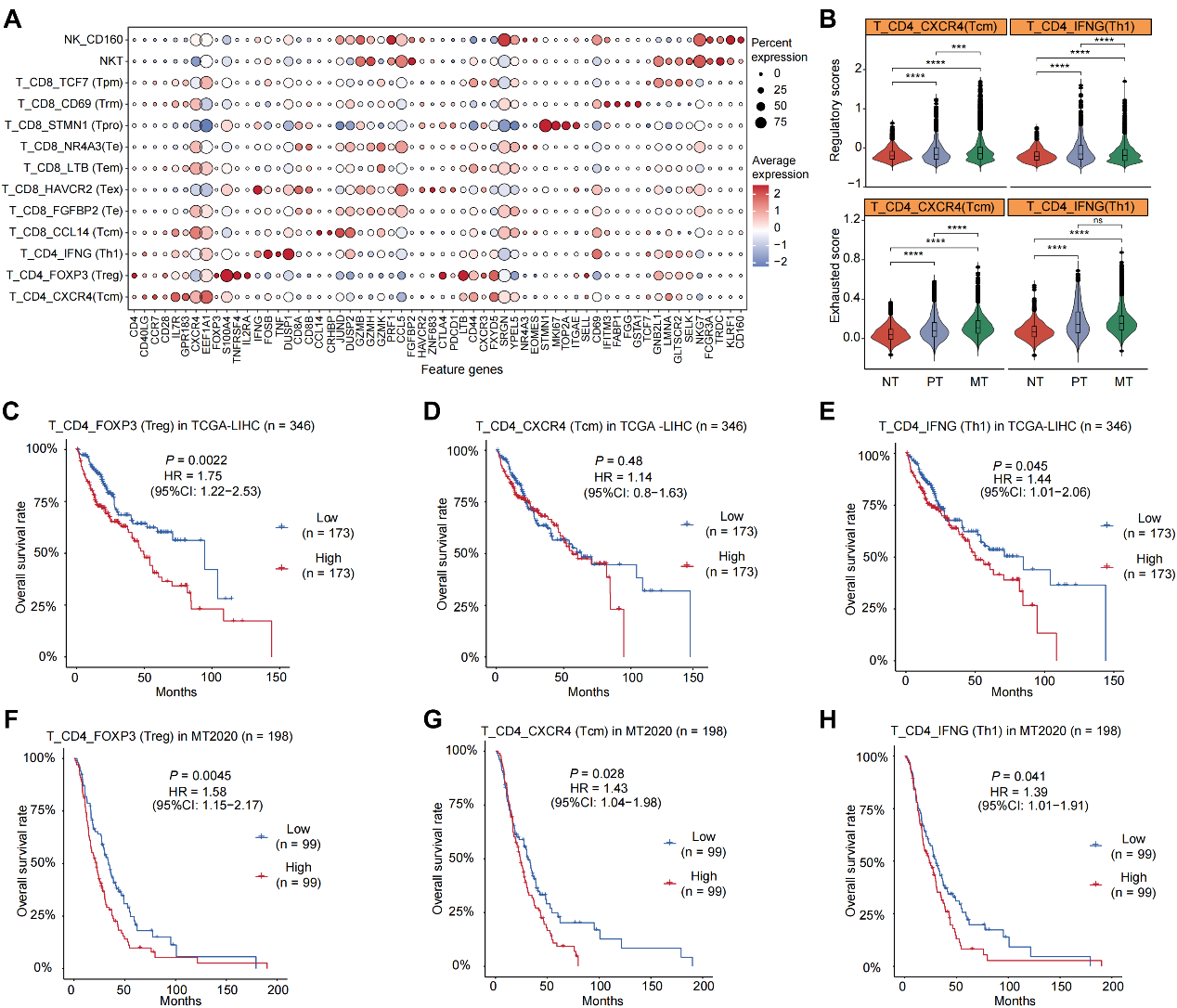


**Fig. S3. Ontological and functional changes of CD4+ T cells in primary and metastatic liver tumors. (A)** Dotplot showing the percentage of expressed cells and average expression levels of marker genes of 13 T/NK cell clusters. **(B)** Violin plots showing the regulatory (top) and exhausted (bottom) scores of CD4+ T cell clusters (except T_CD4_FOXP3 [Treg]) across different types of tissues. Wilcox test was used to assess the difference between groups. “****”, “***” and “ns” represent “*P* < 0.0001”, “*P* < 0.001” and “not significant”, respectively. **(C-E)** Kaplan-Meier survival plots of patients with primary hepatocellular carcinoma from the TCGA-LIHC cohort (n = 346) grouped by the signature scores of each CD4+ T cell cluster. The cluster infiltration levels were estimated based on the top 50 signature genes of each cell cluster by GSVA. The median GSVA score was used to categorize patients into “high” and “low” groups. **(F-H)** Kaplan-Meier survival plots of patients with metastatic liver tumors from the MT2020 cohort (n = 198) grouped by the signature scores of each CD4+ T cell cluster. The cluster infiltration levels were estimated based on the top 50 signature genes of each cell cluster by GSVA. The median GSVA score was used to categorize patients into “high” and “low” groups. GSVA, gene set variation analysis; TCGA-LIHC, The Cancer Genome Atlas-liver hepatocellular carcinoma.


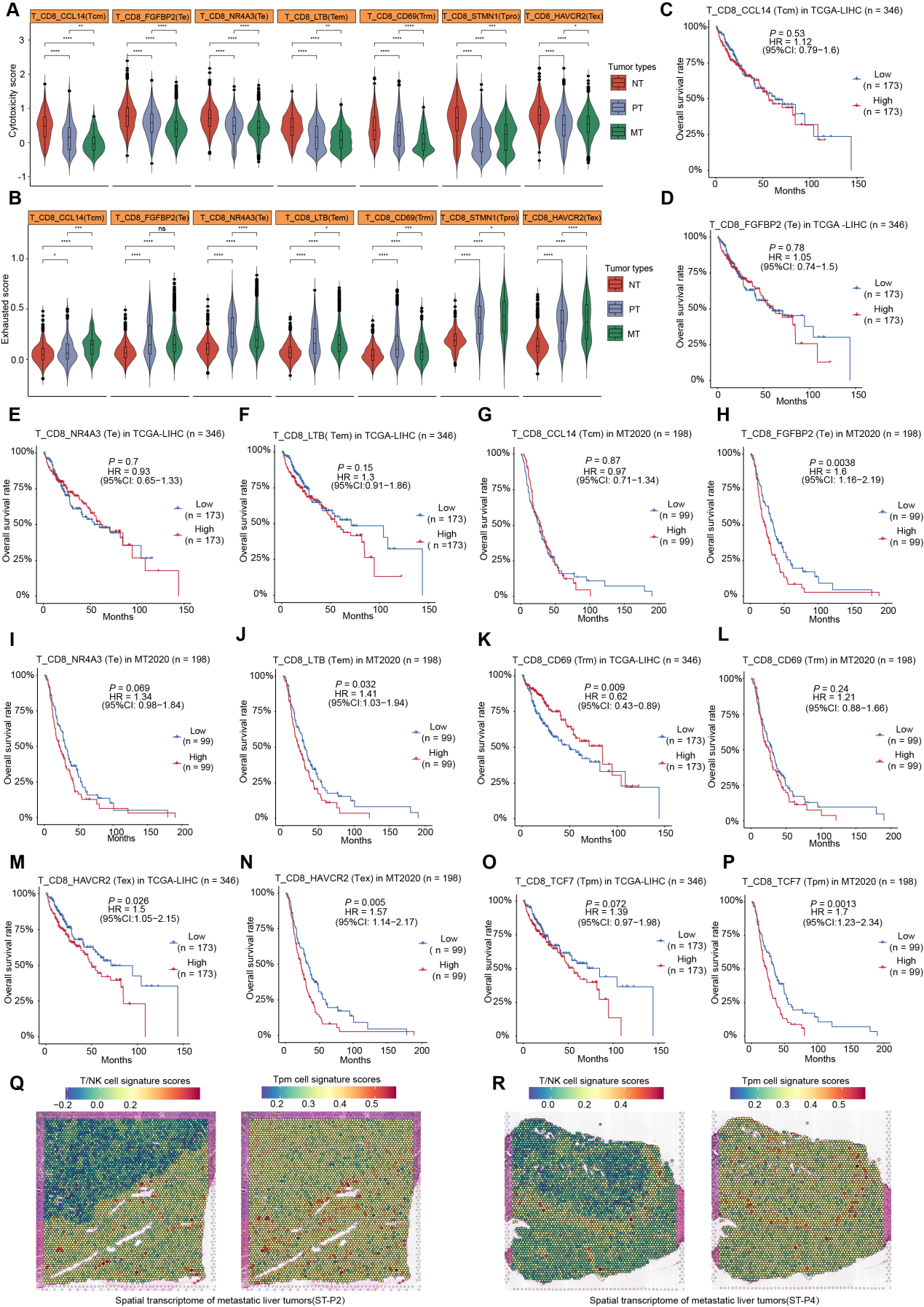


**Fig. S4.** **Functional changes and clinical relevance of CD8+ T cell signatures in primary and metastatic liver tumors. (A)** Violin plots showing the cytotoxicity scores of CD8+ T cell clusters (except for T_CD8_TCF7 [Tpm]) across different types of tissues. Wilcox test was used to assess the difference between groups. “****”, “***”, “**”, “*” and “ns” represent “*P* < 0.0001”, “*P* < 0.001”, “*P* < 0.01”, “*P* < 0.05” and “not significant”, respectively. **(B)** Violin plots showing the exhausted scores of CD8+ T cell clusters (except for T_CD8_TCF7 [Tpm]) across different types of tissues. Wilcox test was used to assess the difference between groups. “****”, “***”, “*” and “ns” represent “*P* < 0.0001”, “*P* < 0.001”, “*P* < 0.05” and “not significant”, respectively. **(C-F)** Kaplan-Meier survival plots of patients with primary hepatocellular carcinoma from the TCGA-LIHC cohort (n = 346) grouped by the signature scores of each effector, effector memory, central memory CD8+ T cell cluster. The cluster infiltration levels were estimated based on the top 50 signature genes of each cell cluster by GSVA. The median GSVA score was used to categorize patients into “high” and “low” groups. **(G-J)** Kaplan-Meier survival plots of patients with metastatic liver tumors from the MT2020 cohort (n = 198) grouped by the signature scores of each effector, effector memory, central memory CD8+T cell cluster. The cluster infiltration levels were estimated based on the top 50 signature genes of each cell cluster by GSVA. The median GSVA score was used to categorize patients into “high” and “low” groups. **(K-L)** Kaplan-Meier survival plots of patients with primary hepatocellular carcinoma from the TCGA-LIHC cohort (n = 346) and patients with metastatic liver tumors from the MT2020 public cohort (n = 198) grouped by the signature scores of tissue-resident memory CD8+ T cell cluster. The cluster infiltration levels were estimated based on the top 50 signature genes of each cell cluster by GSVA. The median GSVA score was used to categorize patients into “high” and “low” groups. **(M-N)** Kaplan-Meier survival plots of patients with primary hepatocellular carcinoma from the TCGA-LIHC cohort (n = 346) and patients with metastatic liver tumors from the MT2020 cohort (n = 198) grouped by the signature scores of exhausted CD8+ T cell cluster. The cluster infiltration levels were estimated based on the top 50 signature genes of each cell cluster by GSVA. The median GSVA score was used to categorize patients into “high” and “low” groups. **(O-P)** Kaplan-Meier survival plots of patients with primary hepatocellular carcinoma from the TCGA-LIHC cohort (n = 346) and patients with metastatic liver tumors from the MT2020 cohort (n = 198) grouped by the signature scores of progenitor-like memory CD8+ T cell cluster. The cluster infiltration levels were estimated based on the top 50 signature genes of each cell cluster by GSVA. The median GSVA score was used to categorize patients into “high” and “low” groups. **(Q-R)** Spatial distribution of the whole T/NK cells and TCF7+ Tpm cells in two metastatic liver tumors (ST-P2 and ST -P4) determined by the spatial transcriptomic data (PMID: 34417225). GSVA, gene set variation analysis; Tpm, progenitor-like memory CD8+ T cells; TCGA-LIHC, The Cancer Genome Atlas-liver hepatocellular carcinoma.


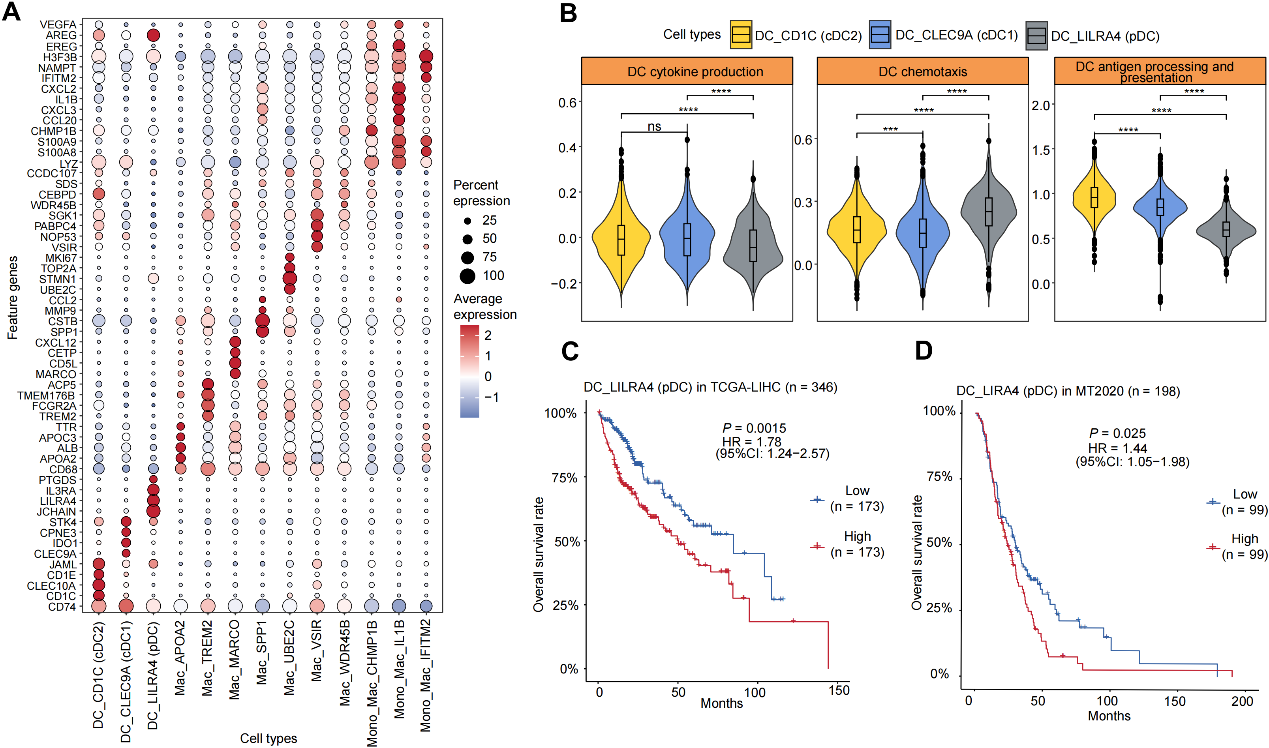


**Fig. S5. Characteristics of myeloid cells in primary and metastatic liver tumors. (A)** Dotplot showing the percentage of expressed cells and average expression levels of marker genes of 13 myeloid cell clusters. **(B)** Violin plots showing the cytokine production (left), chemotaxis (middle), antigen processing and presentation (right) scores of 3 DC clusters. Wilcox test was used to assess the difference between groups. “****”, “***” and “ns” represent “*P* < 0.0001”, “*P* < 0.001” and “not significant”, respectively. **(C)** Kaplan-Meier survival plots of patients with primary hepatocellular carcinoma from the TCGA-LIHC cohort (n = 346) grouped by the signature scores of the pDC cell cluster. The cluster infiltration levels were estimated based on the top 50 signature genes of pDC cell cluster by GSVA. The median GSVA score was used to categorize patients into “high” and “low” groups. **(D)** Kaplan-Meier survival plots of patients with metastatic liver tumors from the MT2020 cohort (n = 198) grouped by the signature scores of the pDC cell cluster. The cluster infiltration levels were estimated based on the top 50 signature genes of each cell cluster by GSVA. The median GSVA score was used to categorize patients into “high” and “low” groups. DC, dendritic cell; GSVA, gene set variation analysis; TCGA-LIHC, The Cancer Genome Atlas-liver hepatocellular carcinoma.


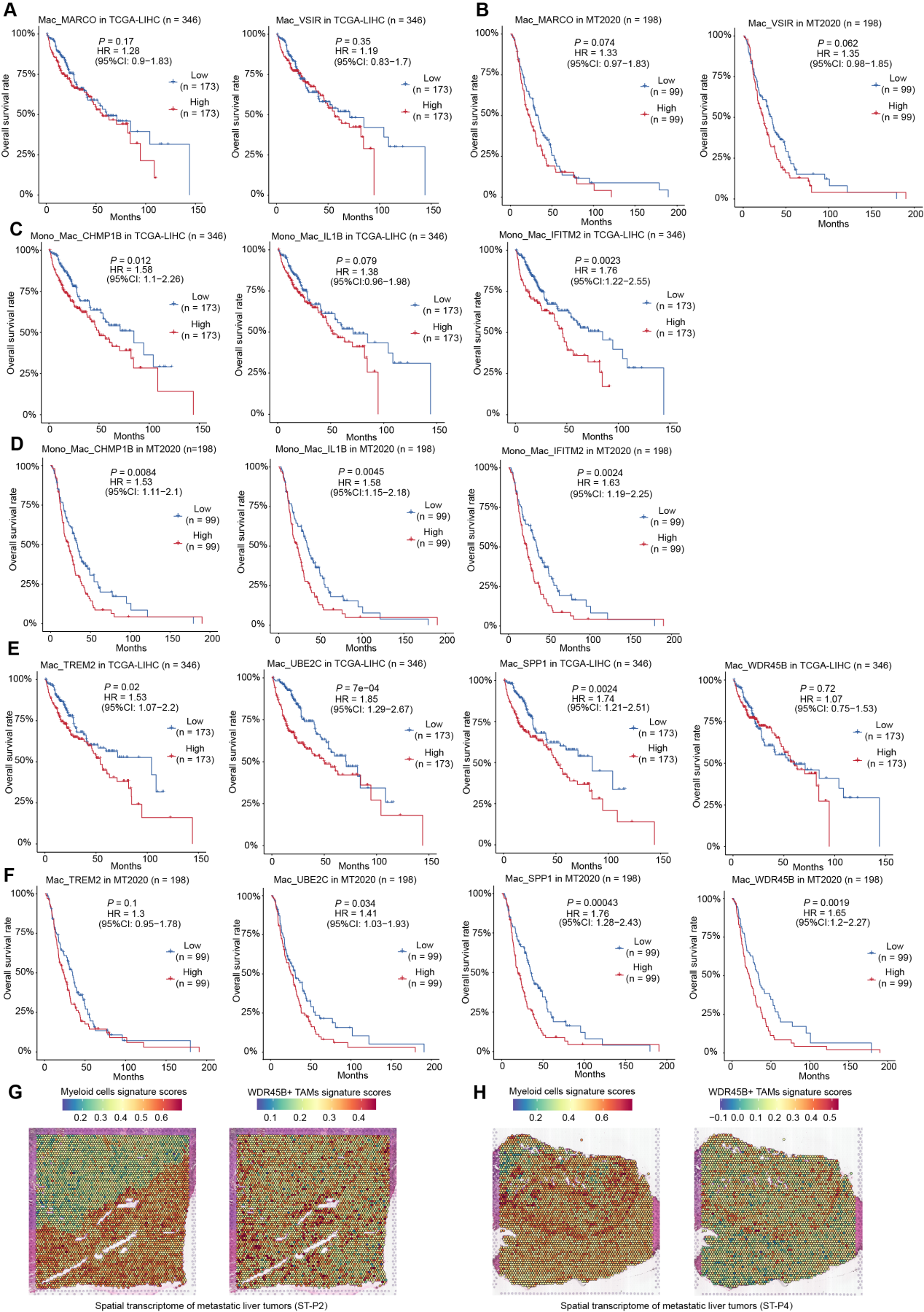


**Fig. S6. Clinical relevance and spatial distribution of macrophage signatures in primary and metastatic liver tumors. (A)** Kaplan-Meier survival plots of patients with primary hepatocellular carcinoma from the TCGA-LIHC cohort (n = 346) grouped by the signature scores of liver-resident Kupffer cells and MHC+ macrophages. The cluster infiltration levels were estimated based on the top 50 signature genes of each cell cluster by GSVA. The median GSVA score was used to categorize patients into “high” and “low” groups. **(B)** Kaplan-Meier survival plots of patients with metastatic liver tumors from the MT2020 cohort (n = 198) grouped by the signature scores of liver-resident Kupffer cells and MHC+ macrophages. The cluster infiltration levels were estimated based on the top 50 signature genes of each cell cluster by GSVA. The median GSVA score was used to categorize patients into “high” and “low” groups. **(C)** Kaplan-Meier survival plots of patients with primary hepatocellular carcinoma from the TCGA-LIHC cohort (n = 346) grouped by the signature scores of each MOMFs cluster. The cluster infiltration levels were estimated based on the top 50 signature genes of each cell cluster by GSVA. The median GSVA score was used to categorize patients into “high” and “low” groups. **(D)** Kaplan-Meier survival plots of patients with metastatic liver tumors from the MT2020 cohort (n = 198) grouped by the signature scores of each MOMFs cluster. The cluster infiltration levels were estimated based on the top 50 signature genes of each cell cluster by GSVA. The median GSVA score was used to categorize patients into “high” and “low” groups. **(E)** Kaplan-Meier survival plots of patients with primary hepatocellular carcinoma from the TCGA-LIHC cohort (n = 346) grouped by the signature scores of each TAMs cluster. The cluster infiltration levels were estimated based on the top 50 signature genes of each cell cluster by GSVA. The median GSVA score was used to categorize patients into “high” and “low” groups. **(F)** Kaplan-Meier survival plots of patients with metastatic liver tumors from the MT2020 cohort (n = 198) grouped by the signature scores of each TAMs cluster. The cluster infiltration levels were estimated based on the top 50 signature genes of each cell cluster by GSVA. The median GSVA score was used to categorize patients into “high” and “low” groups. **(G-H)** Spatial distribution of the whole myeloid cells and WDR45B+ TAMs in two metastatic liver tumors (SP-T1 and ST-P4) determined by the spatial transcriptomic data (PMID: 34417225). TAM, tumor-associated macrophages; GSVA, gene set variation analysis; MoMF, monocyte-derived inflammatory macrophage; TCGA-LIHC, The Cancer Genome Atlas-liver hepatocellular carcinoma.


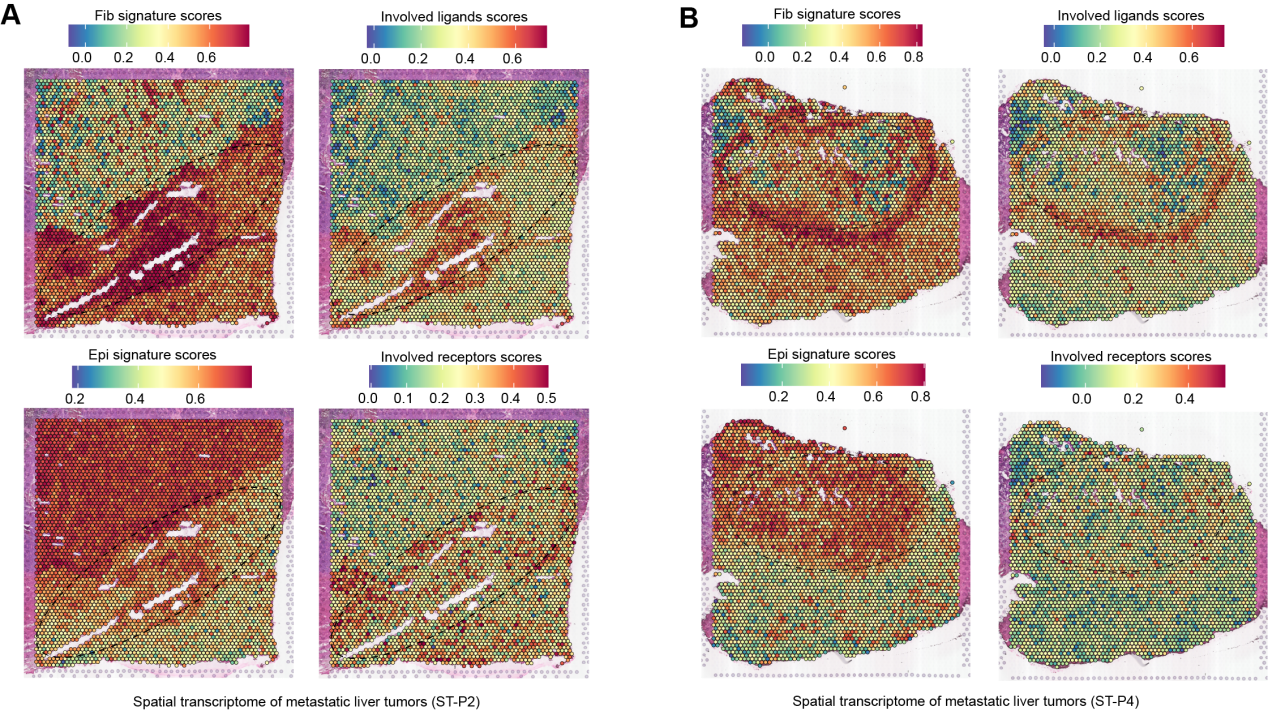


**Fig. S7. Spatial distributions of the fibroblasts, the involved ligands, the malignant epithelial cells and the involved receptors in metastatic liver tumors**. **(A-B)** Spatial distributions of the fibroblasts, the involved ligands, the malignant epithelial cells and the involved receptors in two metastatic liver tumors (ST-P2 and ST-P4) determined by the spatial transcriptomic data (PMID: 34417225).
